# Supplementary material for: Hypergraph-based connectivity measures for signaling pathway topologies
Source: PLoS Comput Biol. 2019 Oct 25;15(10):e1007384. doi: 10.1371/journal.pcbi.1007384 (PMC6834280; doi:10.1371/journal.pcbi.1007384)
Supplement: S1 Fig — Heatmaps showing the effect of filtering pathway representations by blacklisted nodes and small molecules. (A) The proportion of nodes |B≤k| in the Bk-connected set from each source node (rows) for values of k (columns) in the hypergraph. (B) Directed graph connectivity, bipartite graph connectivity, and hypergraph B-connectivity for representations with blacklisted nodes removed. (C) Directed graph connectivity, bipartite graph connectivity, and hypergraph B-connectivity for representations with small molecules and three highly-connected entities (cytosolic Ubiquitin, nuclear Ubiquitin, and the Nuclear Pore Complex) removed. (PDF) [file pcbi.1007384.s001.pdf]

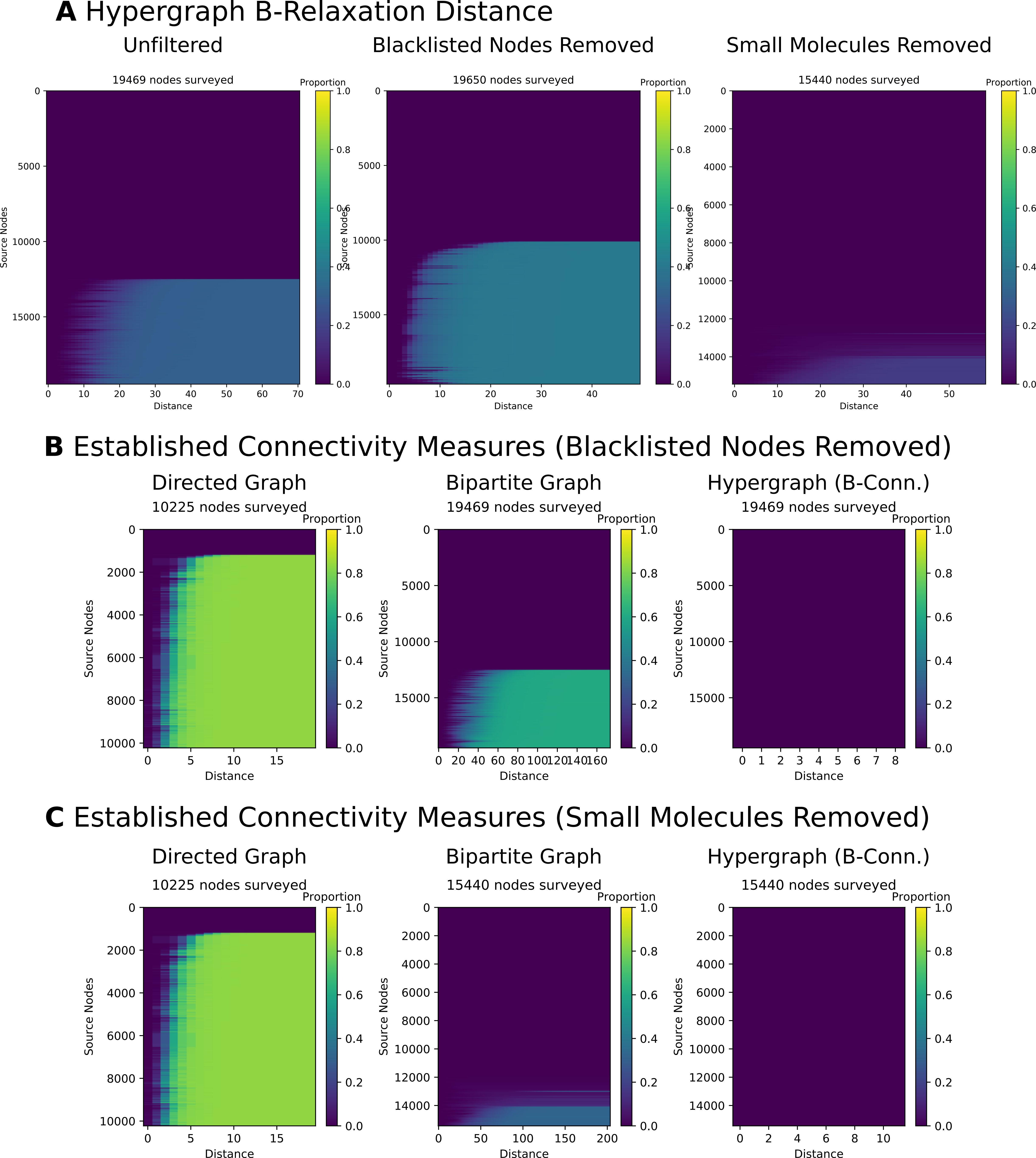
A Hypergraph B-Relaxation Distance
Unfiltered
19469 nodes surveyed
Blacklisted Nodes Removed
19650 nodes surveyed
Small Molecules Removed
15440 nodes surveyed
Source Nodes
Distance
Proportion
B Established Connectivity Measures (Blacklisted Nodes Removed)
Directed Graph
10225 nodes surveyed
Bipartite Graph
19469 nodes surveyed
Hypergraph (B-Conn.)
19469 nodes surveyed
C Established Connectivity Measures (Small Molecules Removed)
Directed Graph
10225 nodes surveyed
Bipartite Graph
15440 nodes surveyed
Hypergraph (B-Conn.)
15440 nodes surveyed
