# Supplementary figures and images for: Hypergraph-based connectivity measures for signaling pathway topologies
Source: PLoS Comput Biol. 2019 Oct 25;15(10):e1007384. doi: 10.1371/journal.pcbi.1007384 (PMC6834280; doi:10.1371/journal.pcbi.1007384)

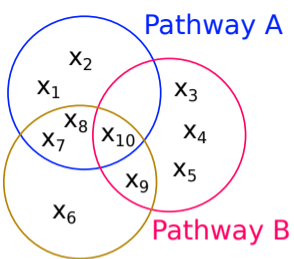

Pathway C

|   | A | B | C |
|---|---|---|---|
| A | 5 | 1 | 3 |
| B | 1 | 5 | 2 |
| C | 3 | 2 | 5 |

$n=10$

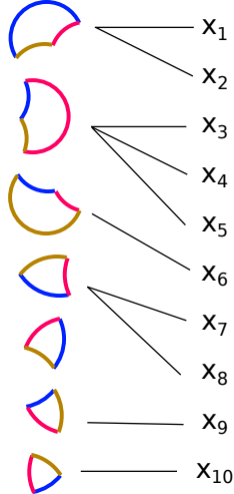

→ Degree-preserving  
edge swaps  
( $m=4$ )

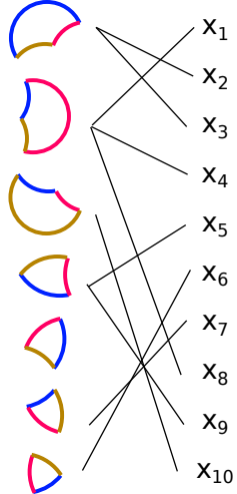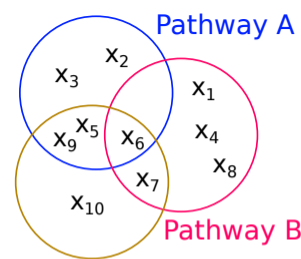

Pathway C

|   | A | B | C |
|---|---|---|---|
| A | 5 | 1 | 3 |
| B | 1 | 5 | 2 |
| C | 3 | 2 | 5 |

$n=10$

Supplement: S2 Fig — The example shows ten molecules (x1, …, x10) that are members of three pathways (A, B and C). The initial pathway overlap, represented as a Venn diagram, results in the matrix of pairwise overlaps (left). We construct an undirected bipartite graph (we’ll call this the permutation graph to distinguish this graph from the bipartite graph representation). In the permutation graph, one set of nodes are the molecules and the other set of nodes are all possible overlapping sets except the null set (here, 2n − 1 = 23 − 1 = 7). Edges in the permutation graph connect molecules to the overlapping set to which they belong. We then perform degree-preserving edge swaps by selecting pairs of edges with different nodes and swapping them (we perform 10,000 swaps in all experiments). We then “piece” the Venn diagram back together, which contains the same number of elements in each overlapping set (resulting in the same pairwise overlaps). With 34 pathways, one would think that the permutation graph is too large; however, we found that there were only 255 non-empty portions of the Venn diagram for the hypergraph/bipartite graph entities (and only 180 for the directed graph entities). The size of the permutation graph also makes the choice of 10,000 swaps for each permutation reasonable. (PDF) [file pcbi.1007384.s002.pdf]

# Directed Graph Influence Scores

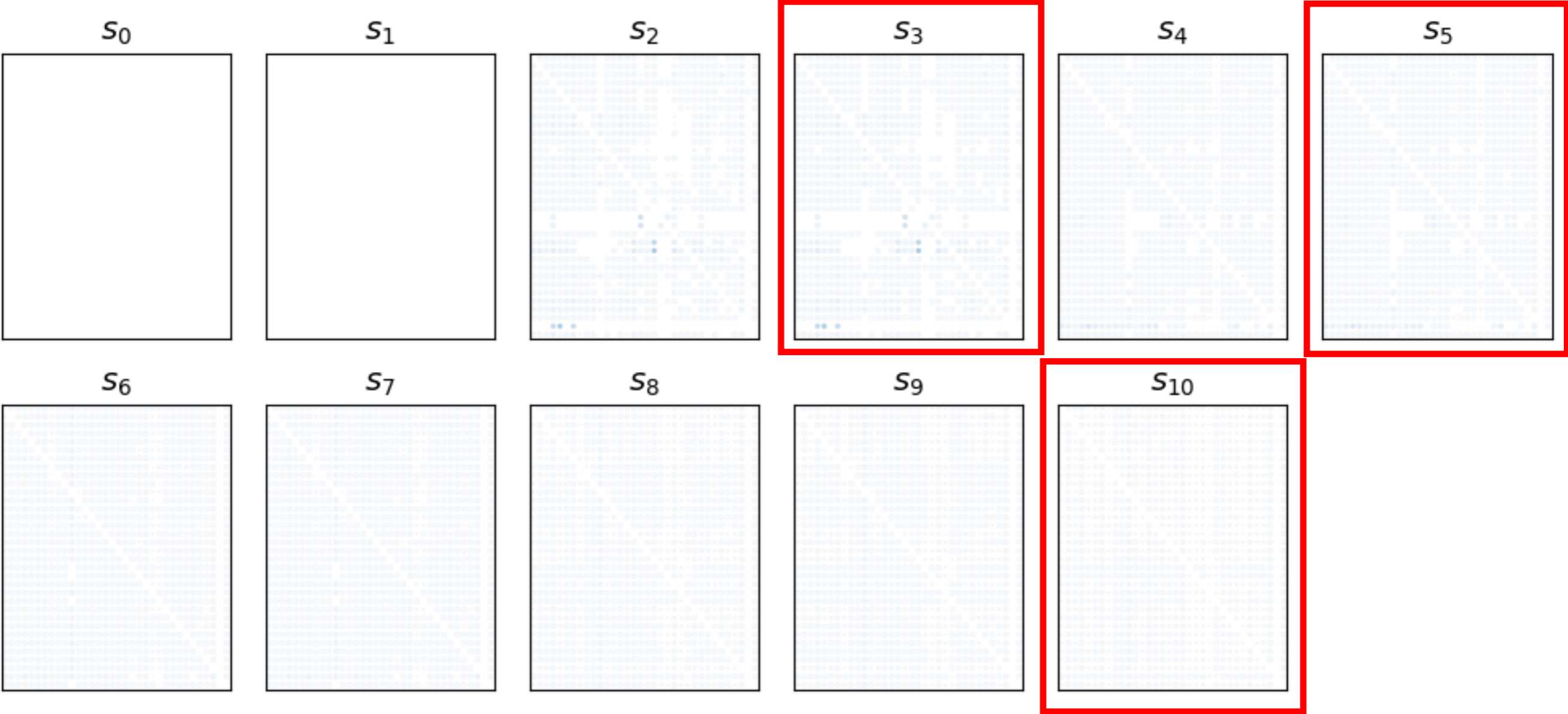

Influence Score  $s_3$

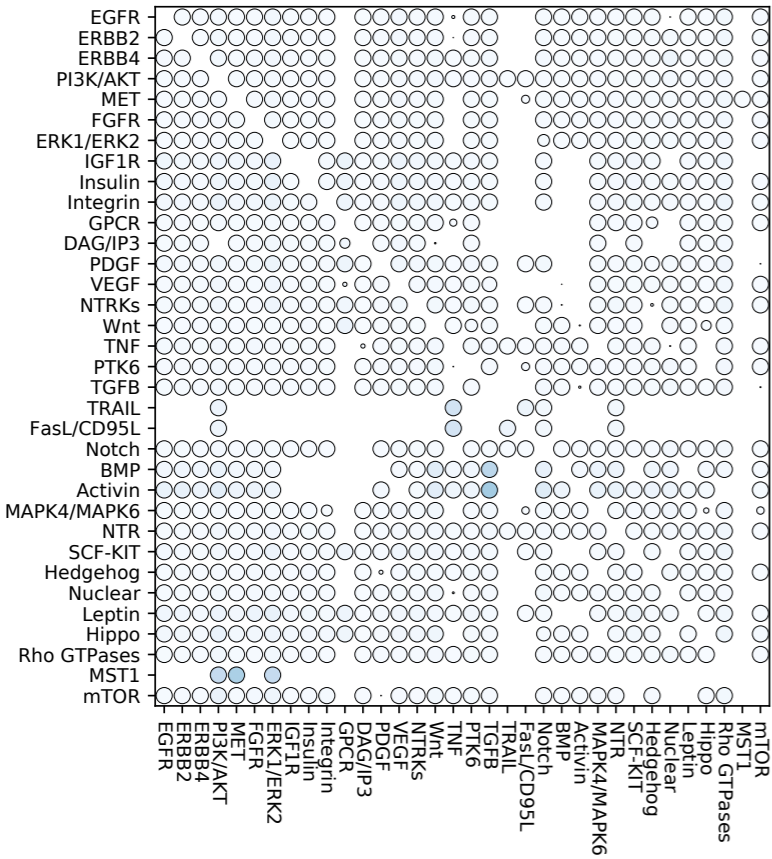

Influence Score  $s_5$

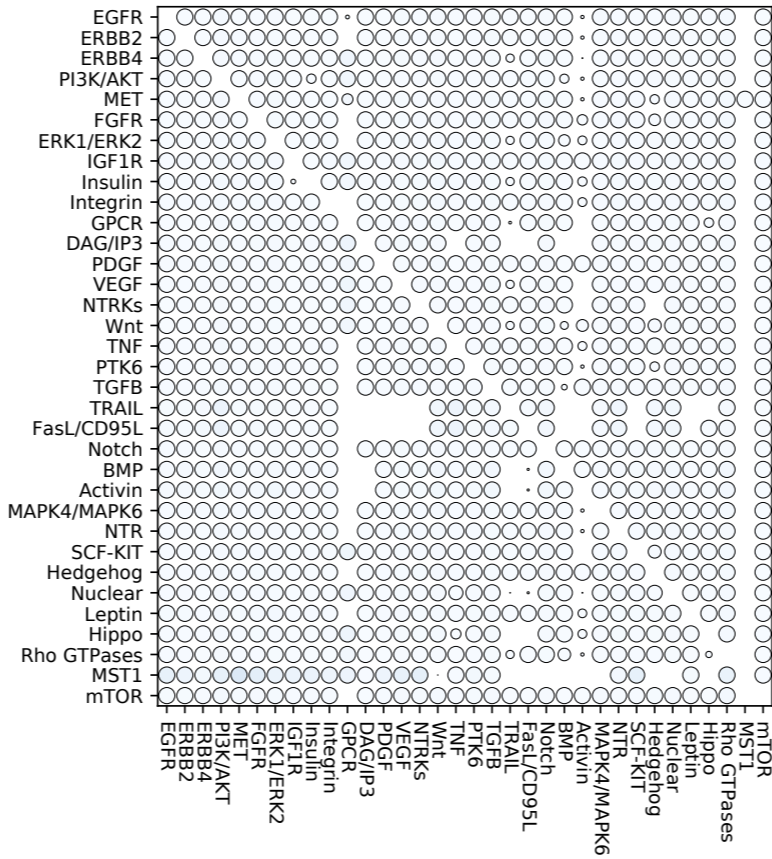

Influence Score  $s_{10}$

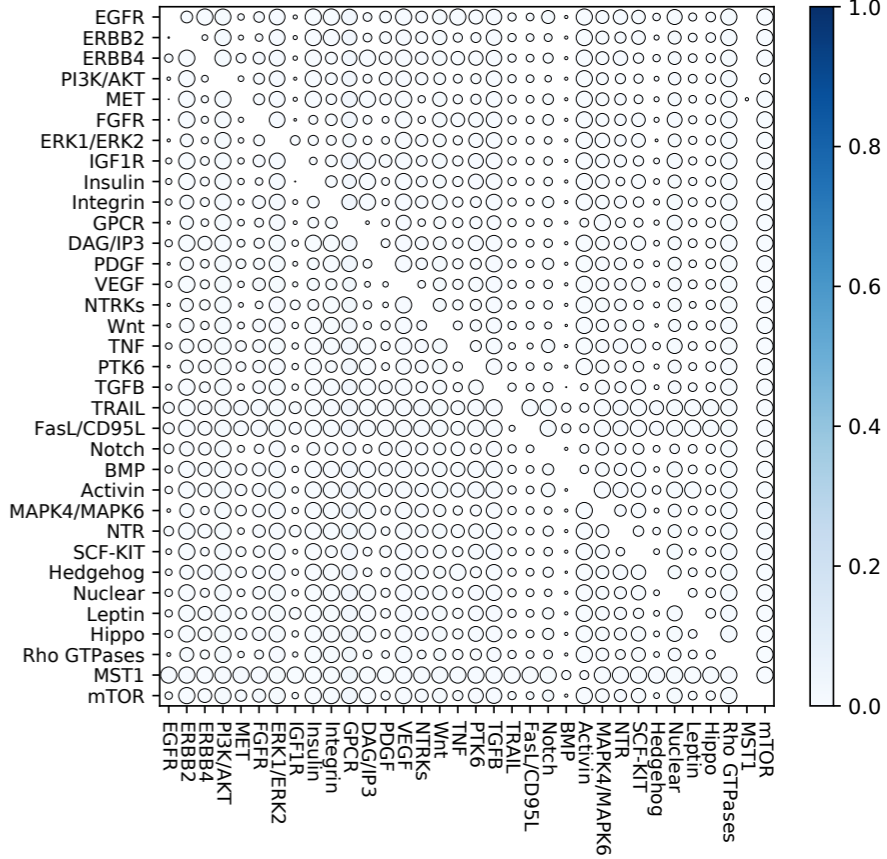

Supplement: S4 Fig — Influence scores of pairs of Reactome pathways for the directed graph at selected values of distance k. Rows indicate the source pathway PS and columns indicated the target pathway PT. Color indicates influence score and circle size indicates significance by permutation test (larger circles are more significant). Three selected distances are enlarged. Note that the entities are different for this graph than the hypergraph and bipartite graph, resulting in different initial pathway overlaps. (PDF) [file pcbi.1007384.s004.pdf]

# Bipartite Graph Influence Scores

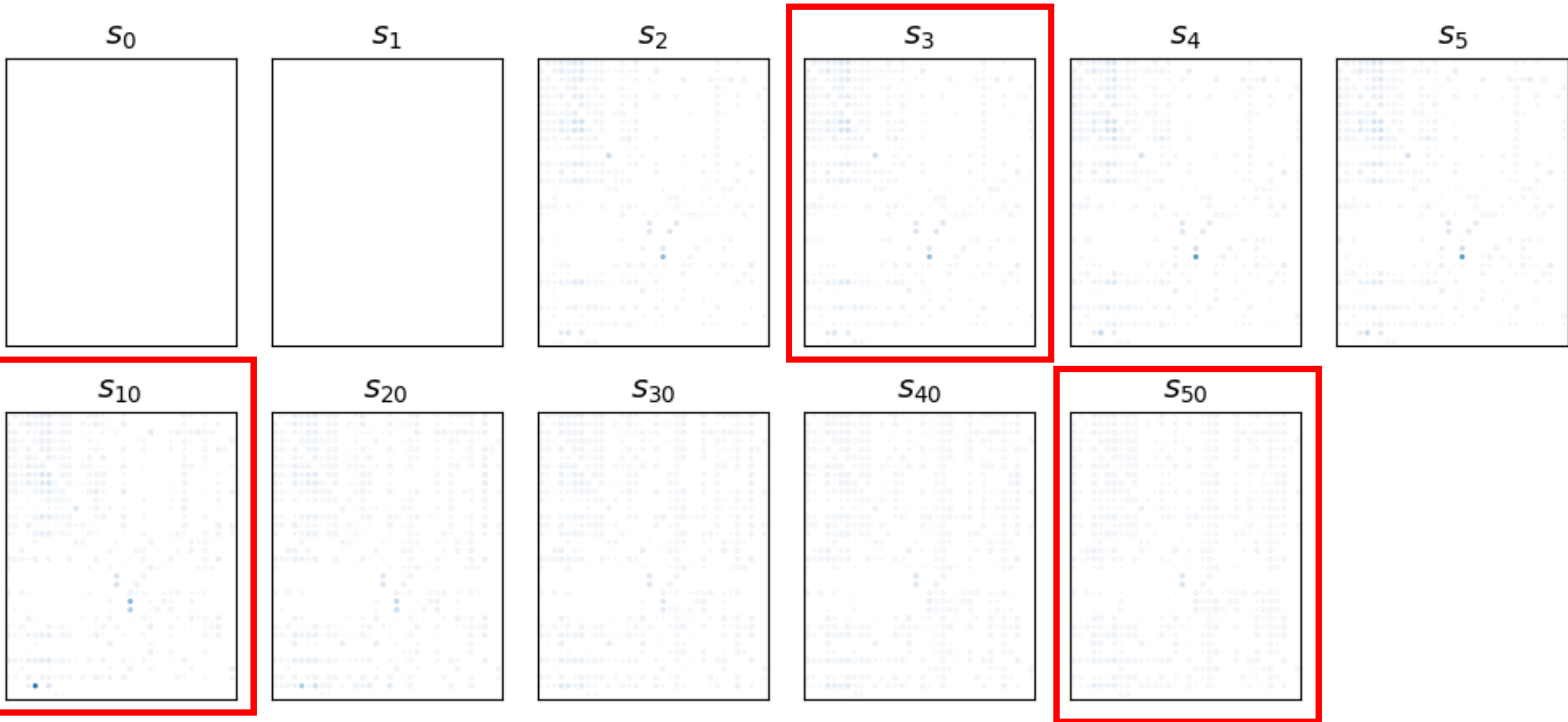

Influence Score  $s_3$

Influence Score  $s_{10}$

Influence Score  $s_{50}$

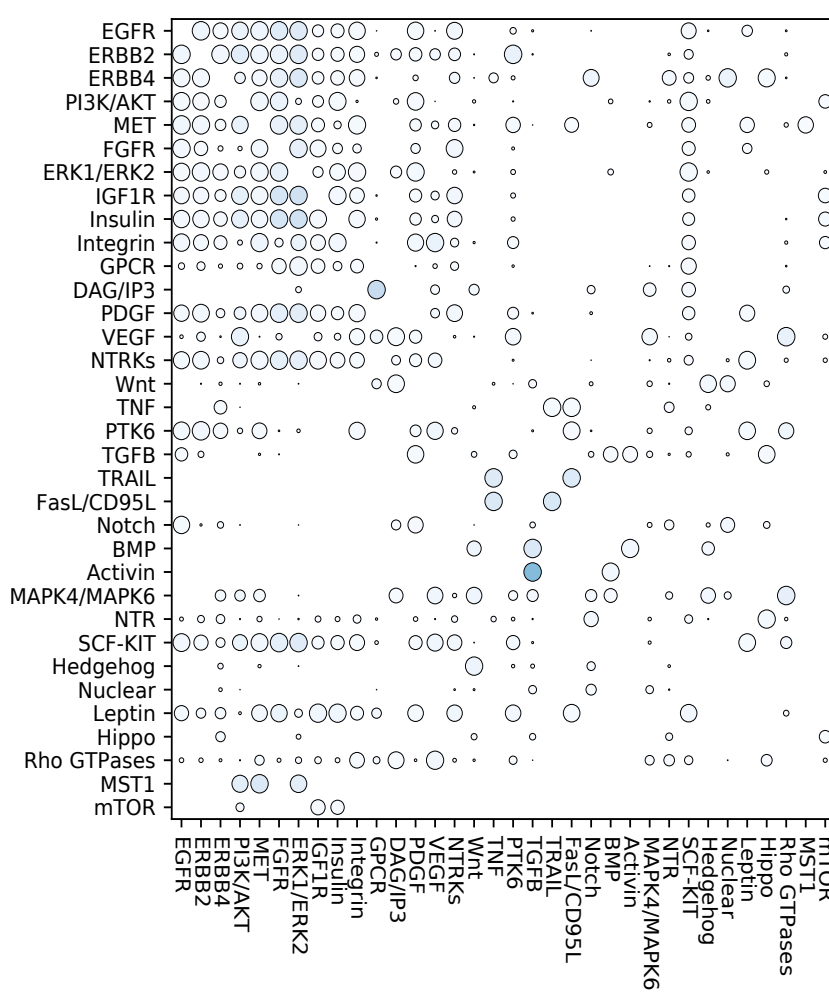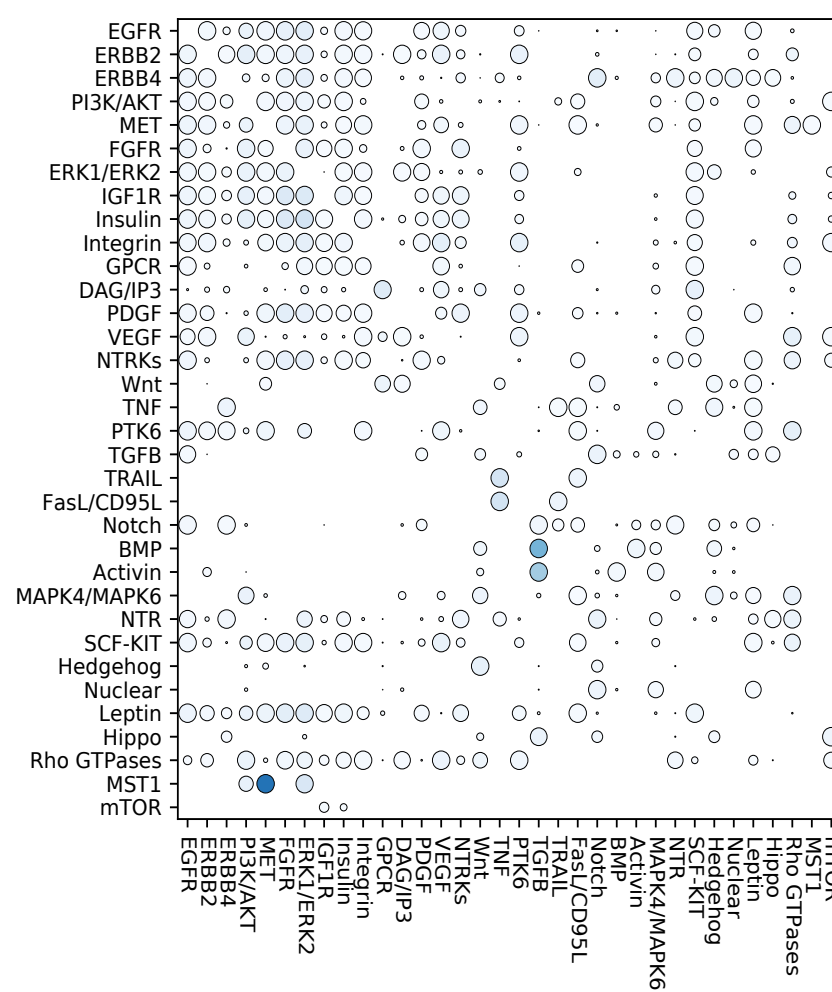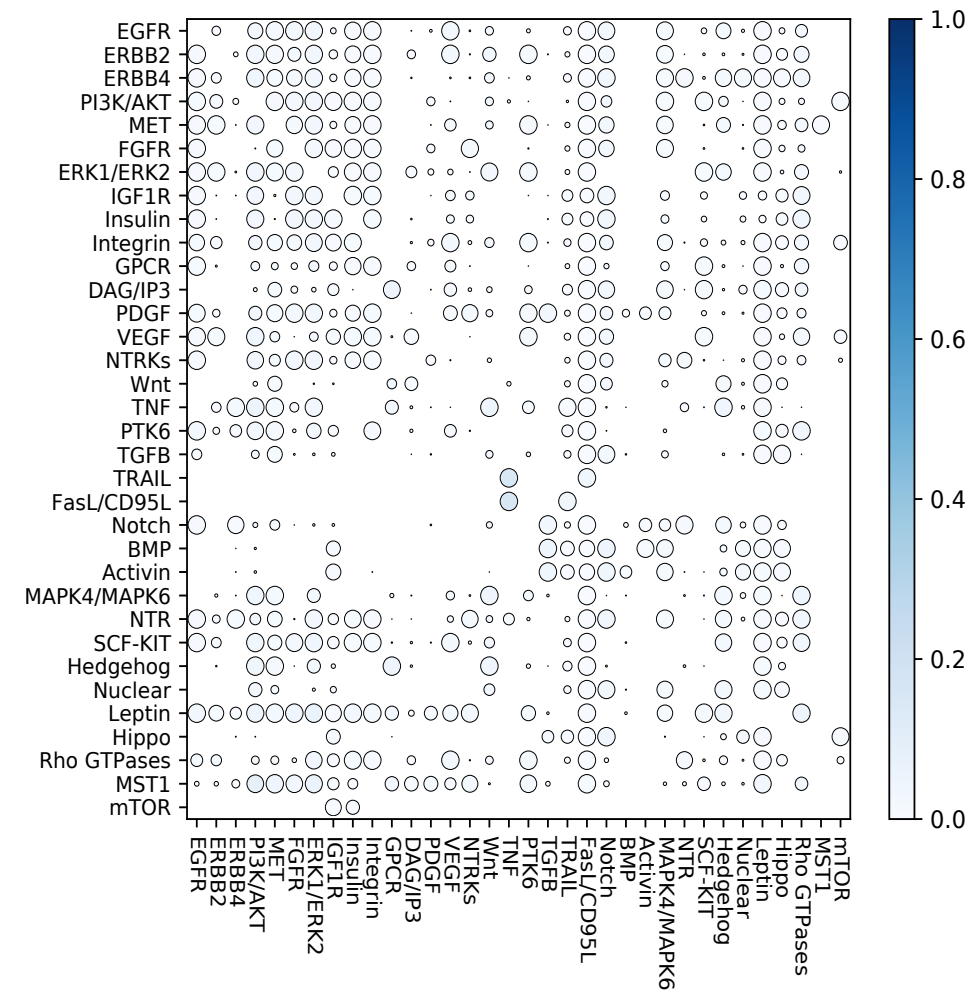

Supplement: S5 Fig — Influence scores of pairs of Reactome pathways for the bipartite graph at selected values of distance k. Rows indicate the source pathway PS and columns indicated the target pathway PT. Color indicates influence score and circle size indicates significance by permutation test (larger circles are more significant). Three selected distances are enlarged. (PDF) [file pcbi.1007384.s005.pdf]

**A**

Source Pathway MST1

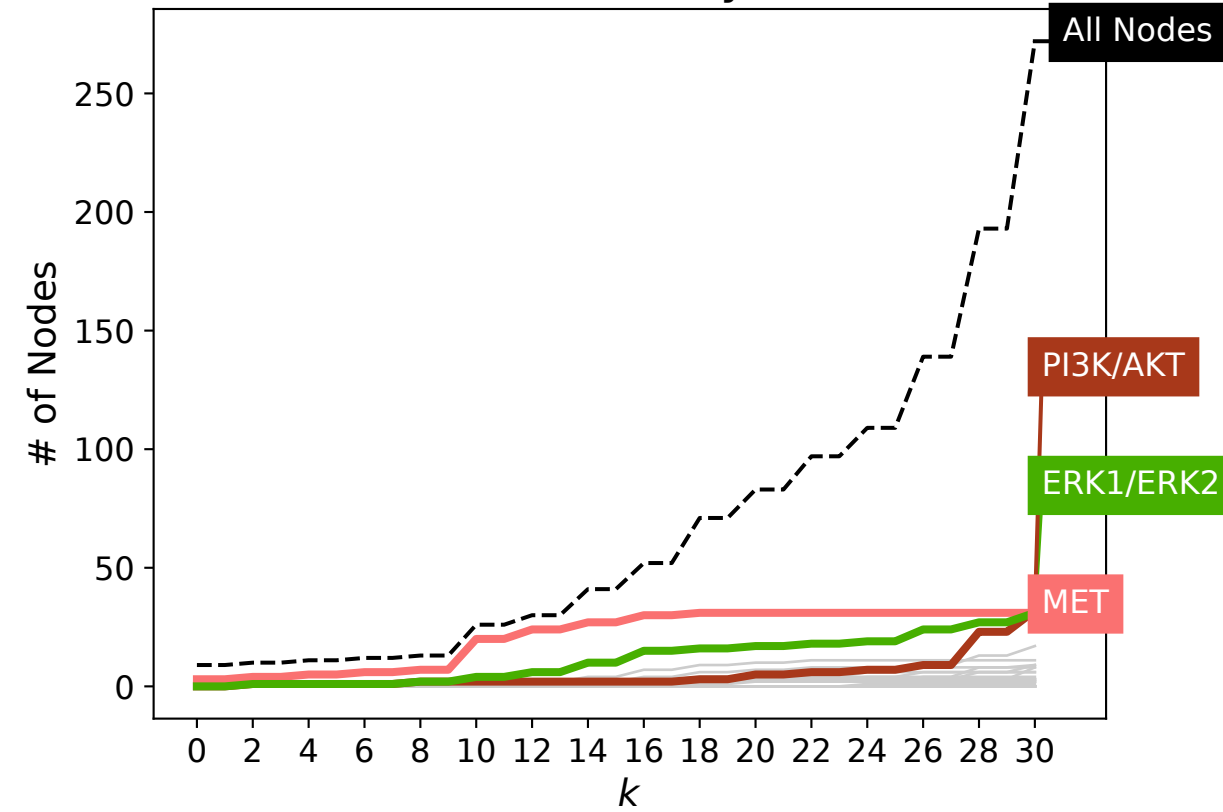**B**

Source Pathway Activin

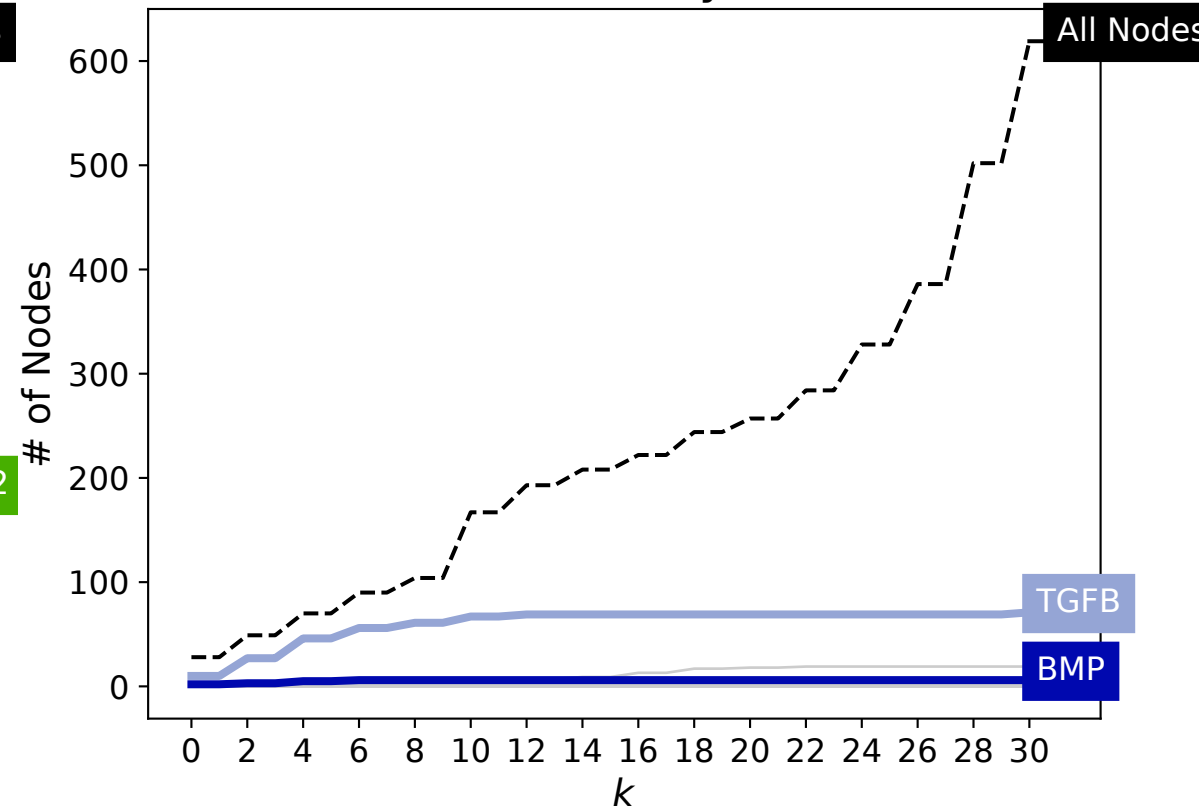**C**

Source Pathway BMP

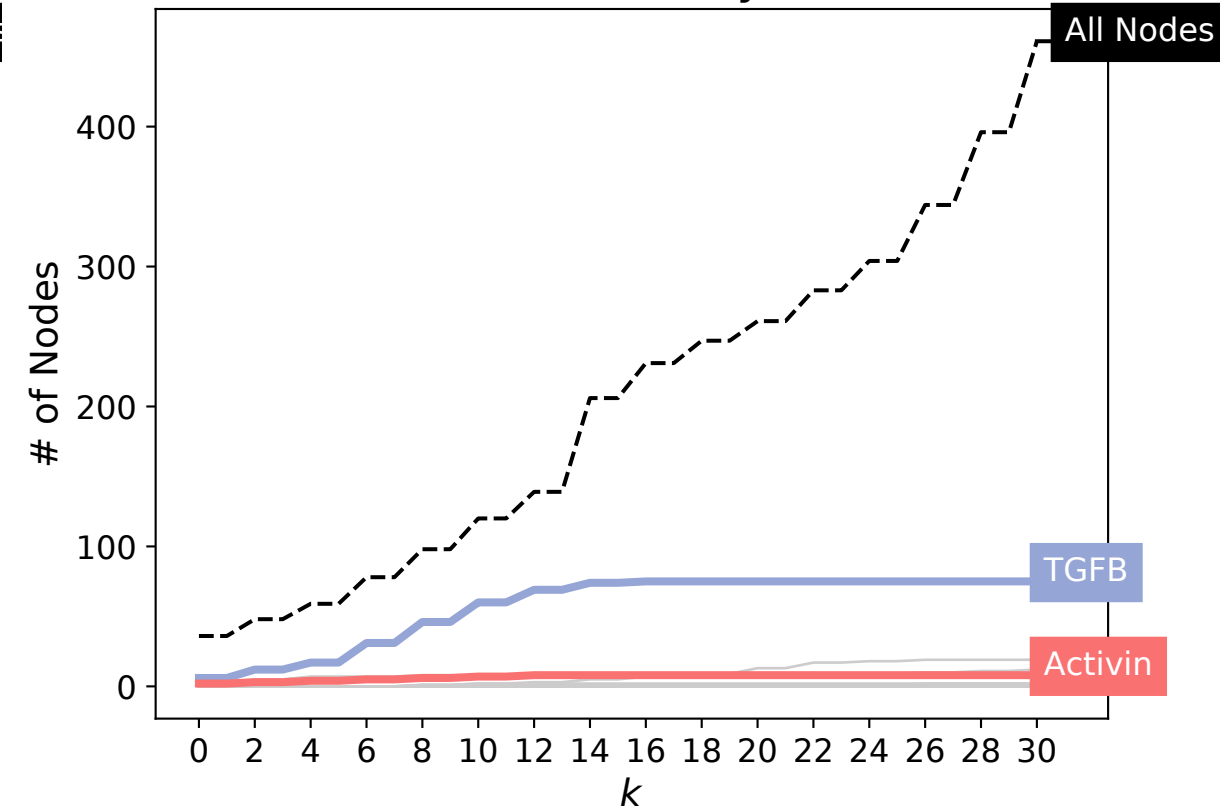

Supplement: S6 Fig — The influence of (A) signaling by Mst1, (B) signaling by BMP, and (C) signaling by Activin on the other Reactome pathways. The dashed black line indicates the number of nodes in the source pathway’s B≤k for different values of k. There is one line for each of the 33 other target pathways denoting the number of members that appear in B≤k, with selected pathways highlighted in bold. (PDF) [file pcbi.1007384.s006.pdf]

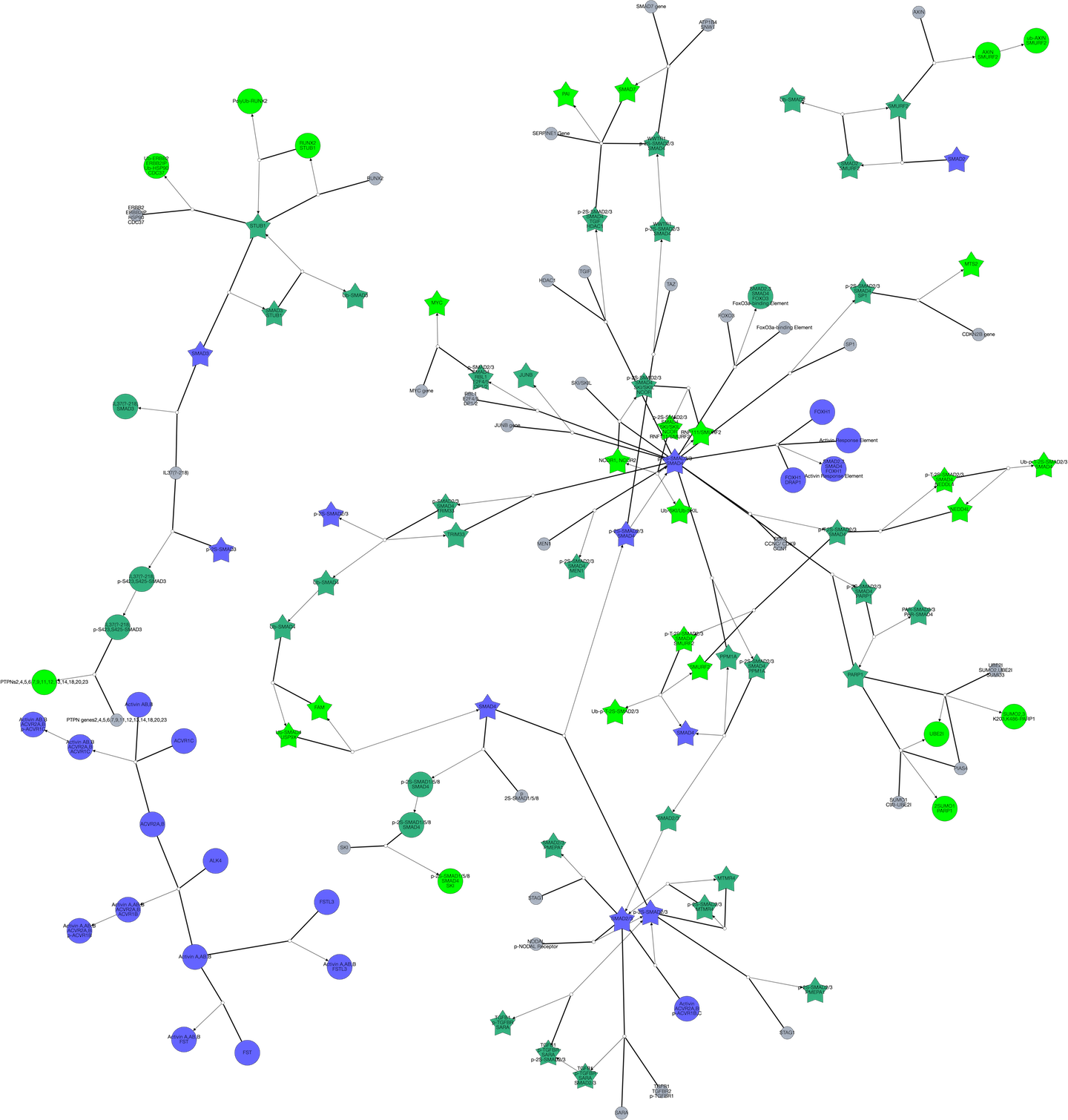

Supplement: S7 Fig — Hyperedges traversed to compute B0, B1, …, B4 from source pathway Activin. Node colors represent B-relaxation distance from k = 0 (B-connected set, blue) to k = 3 (bright green). Gray nodes are entities that are not in the Bk-connected set but are involved in traversed hyperedges. Star-shaped nodes are members of the TGFβ pathway. This network is available on GraphSpace at http://graphspace.org/graphs/26756?user_layout=6713. (PNG) [file pcbi.1007384.s007.png]

# "Experimental" Channel

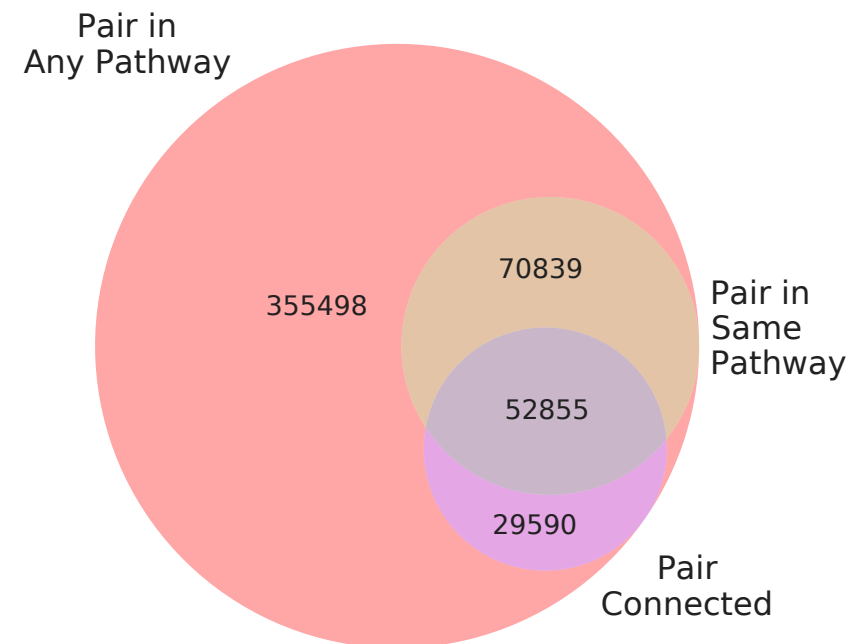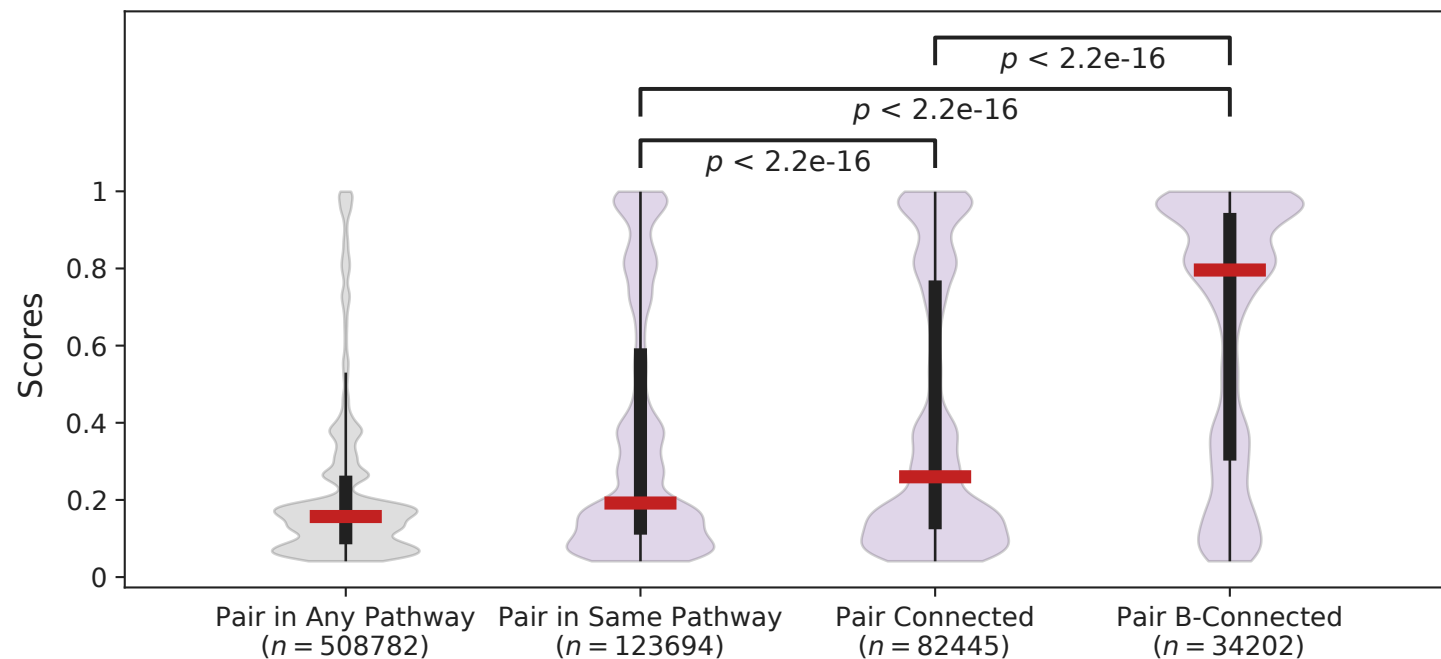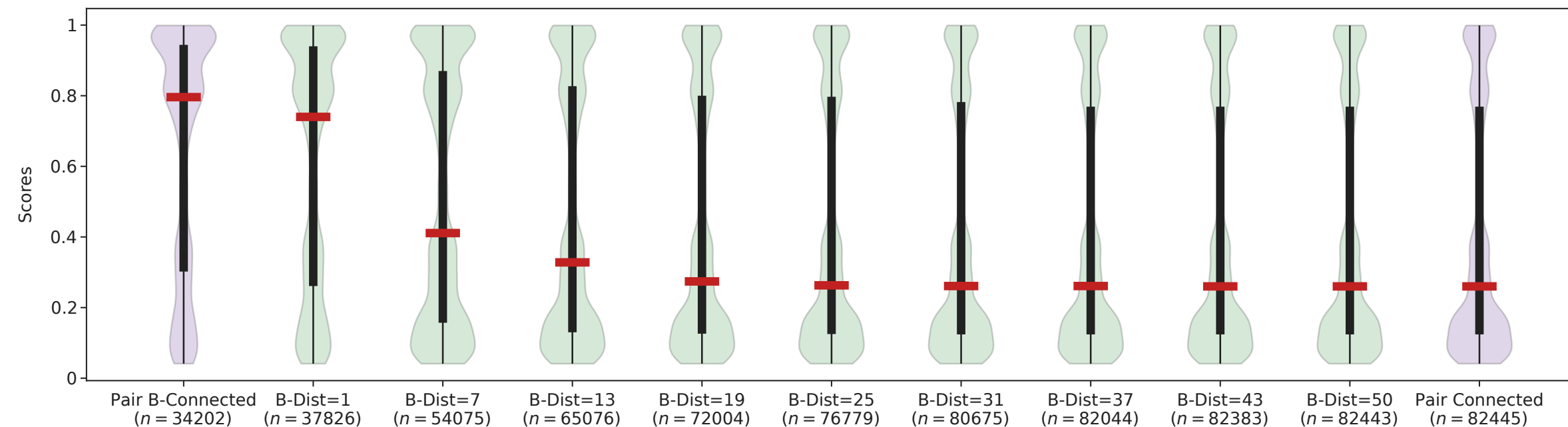

Supplement: S8 Fig — STRING interactions within Reactome for “experimental” interactions. In addition to the components of Fig 7, the bottom violin plot shows the distributions of interaction scores for selected B-relaxation distance thresholds. (PDF) [file pcbi.1007384.s008.pdf]

## "Neighborhood" Channel

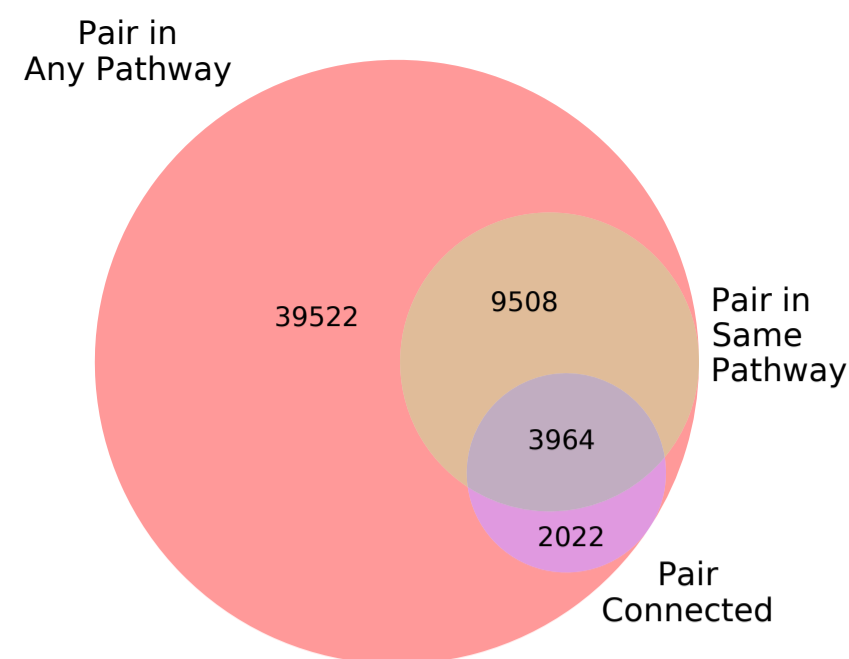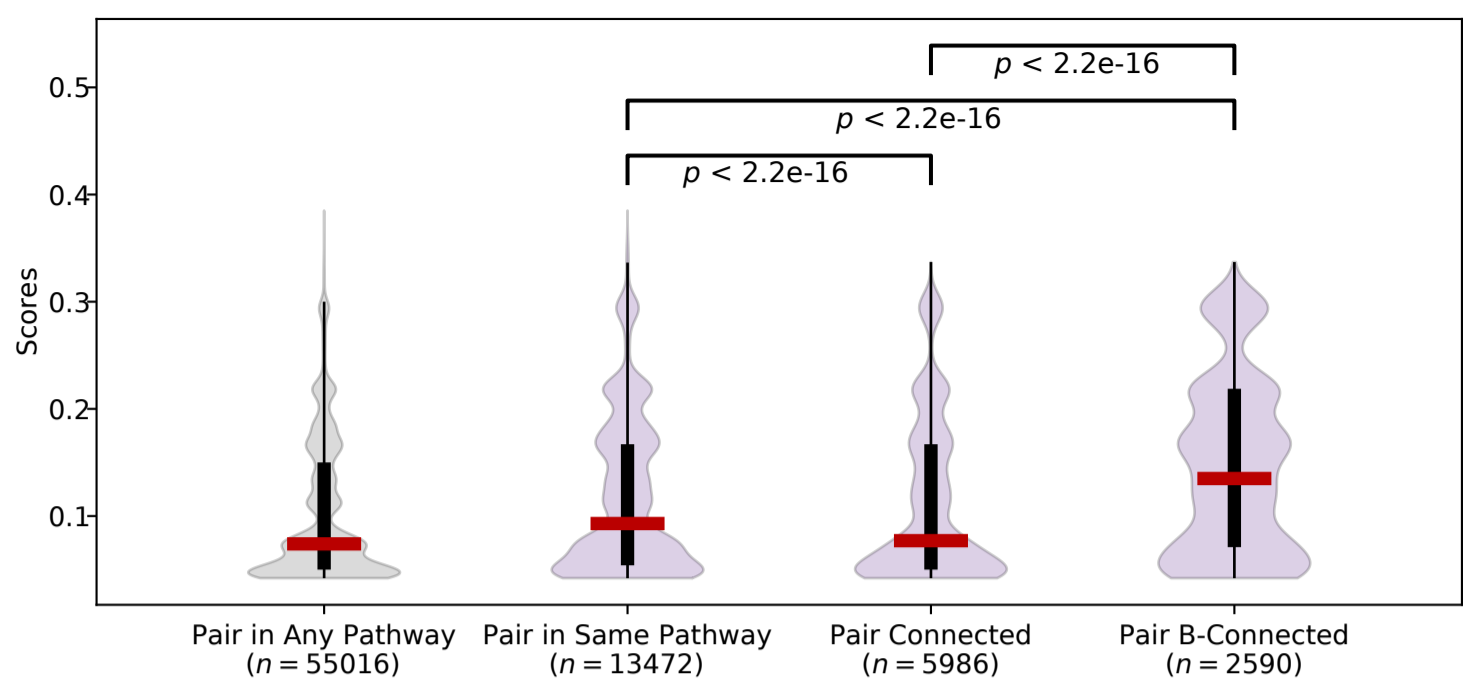

## "Coexpression" Channel

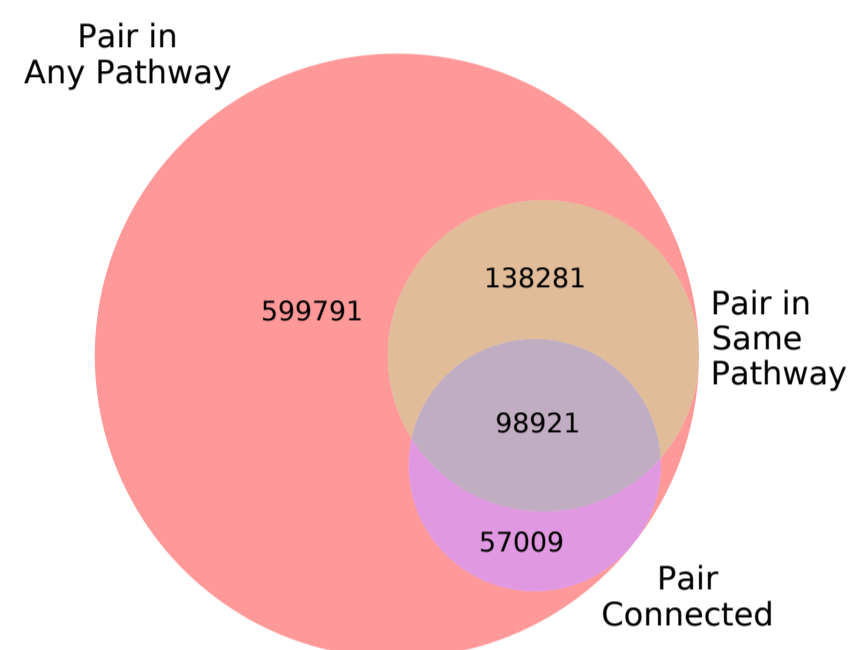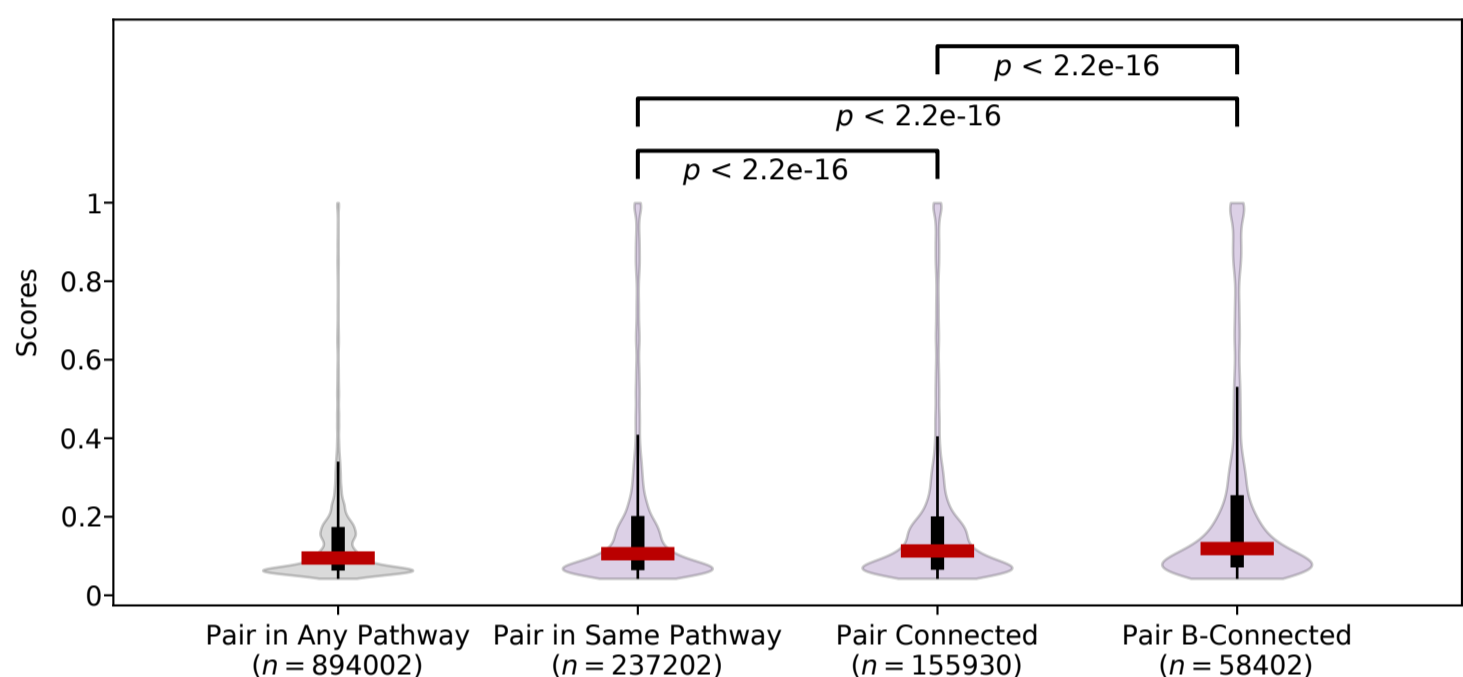

## "Co-occurrence" Channel

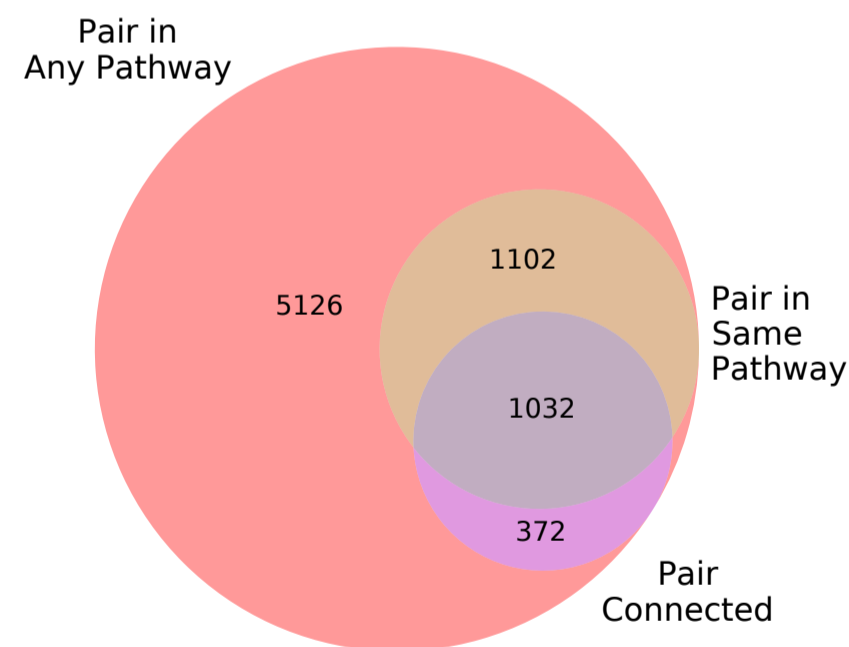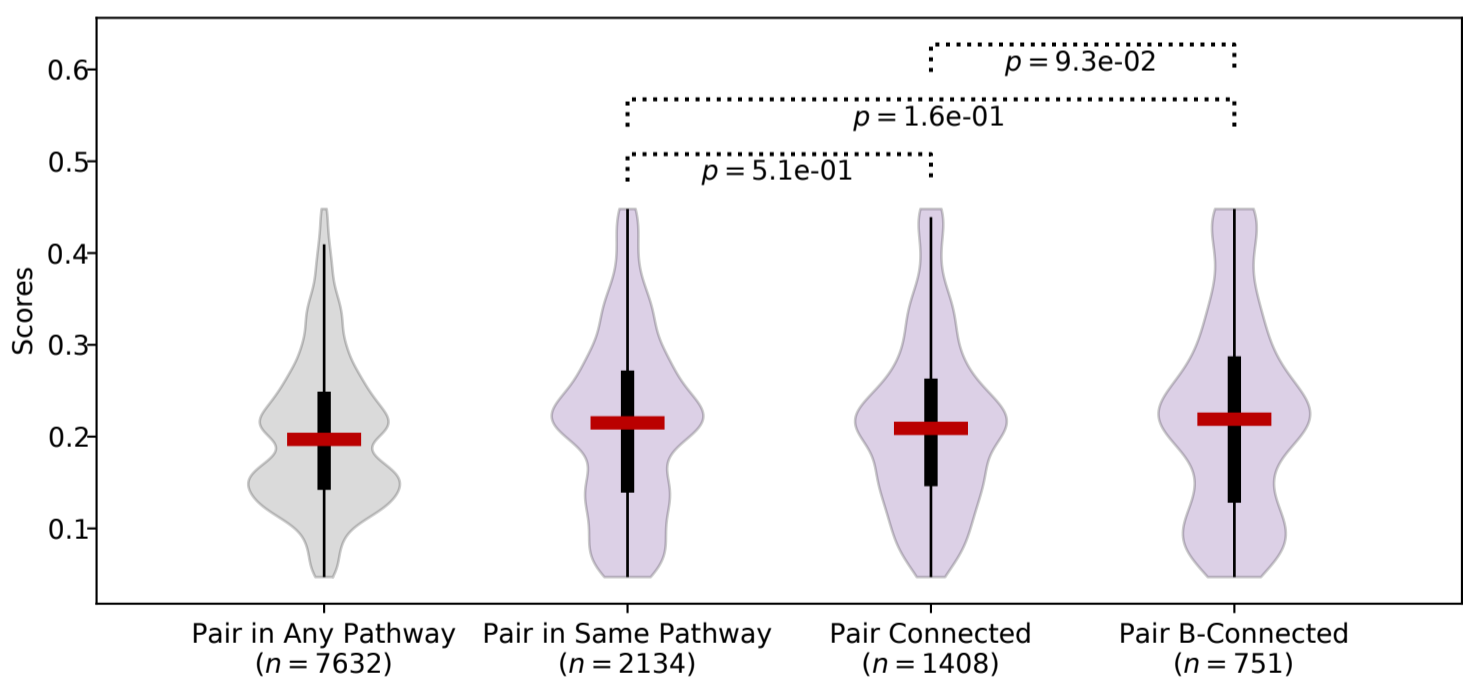

## "Fusion" Channel

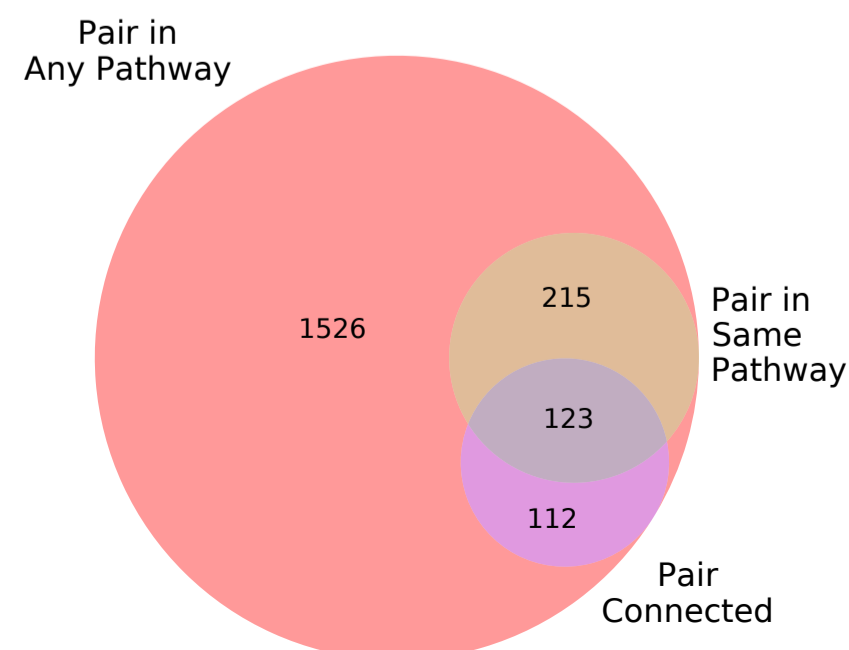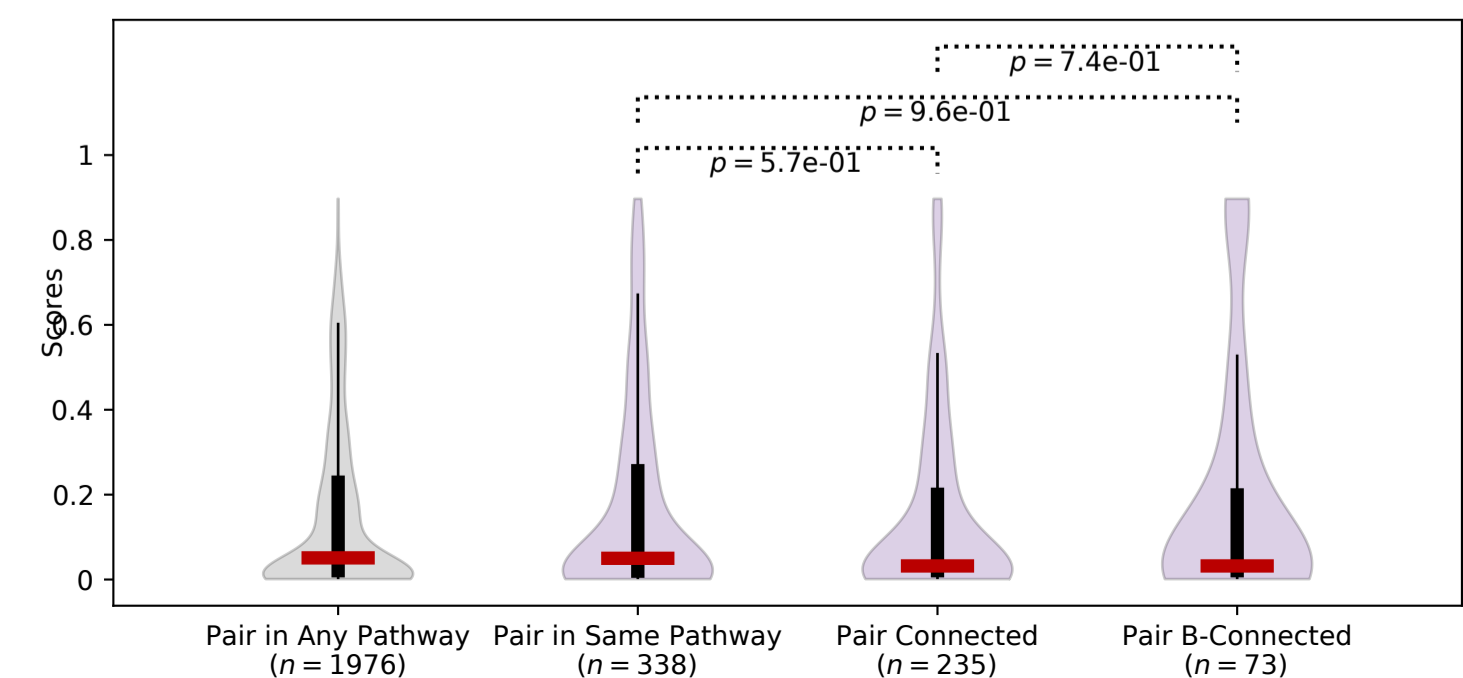

Supplement: S9 Fig — STRING interactions within Reactome for the remaining evidence channels not shown in Fig 7 or S8 Fig. The Venn diagram shows the overlap of interactions where the nodes appear in any Reactome pathway, appear in the same Reactome pathway, or are connected in the bipartite graph. The violin plot shows the distributions of interaction scores for different sets of interactions (median and percentiles shown; Kruskal-Wallis p-values less than 0.01 are shown with a solid line). (PDF) [file pcbi.1007384.s009.pdf]

**Iteration 0 ( $b\_visit()$ )**

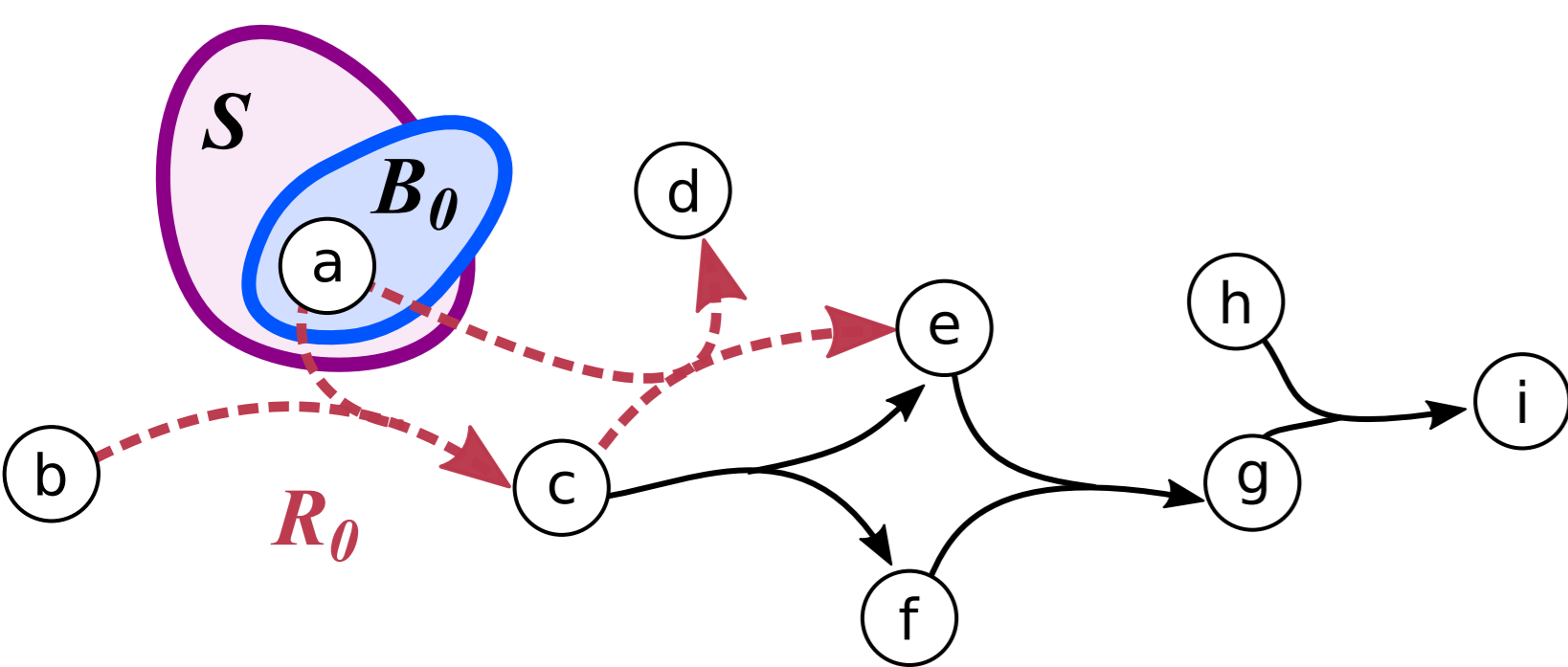

**Iteration 1**

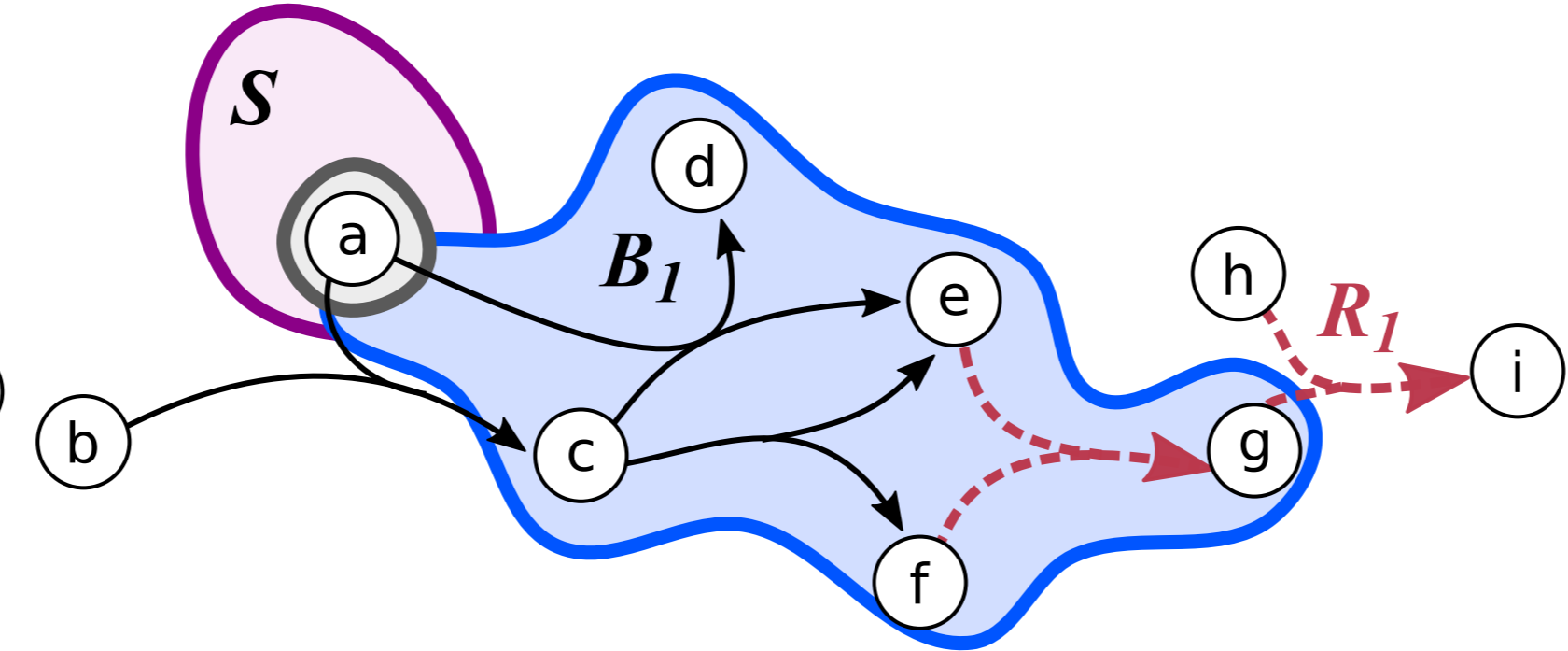

**Iteration 2**

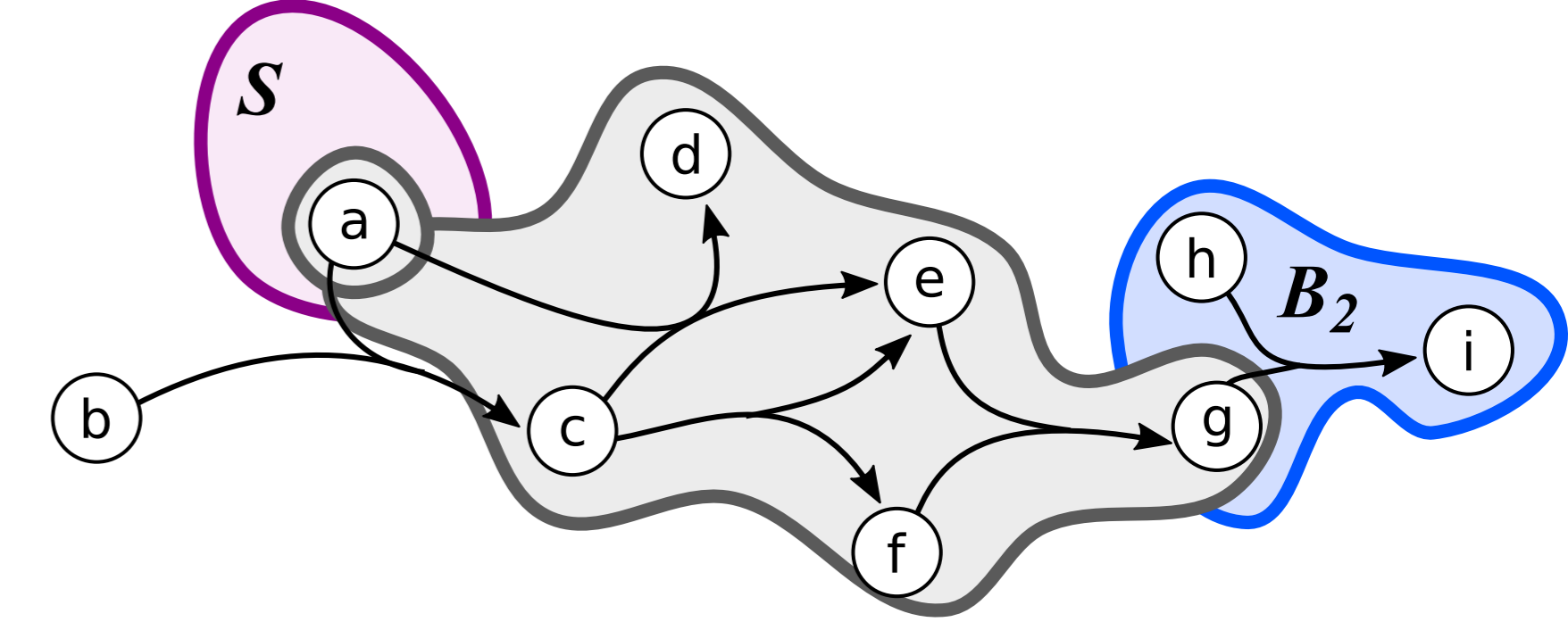

Supplement: S10 Fig — The restrictive set Rk may include hyperedges that have been traversed in a previous iteration’s b_visit() call. In iteration 1, the restrictive set R1 is established by considering the B-connectivity from the heads of the two hyperedges in R0. The hyperedge {{e, f}, {g}} is restrictive with respect to the heads of one hyperedge in R0 but traversable with respect to the heads of the other hyperedge. Thus, {{e, f}, {g}} is included in R1 but also added to the seen dictionary, saving redundant computation in Algorithm 2. (PDF) [file pcbi.1007384.s010.pdf]

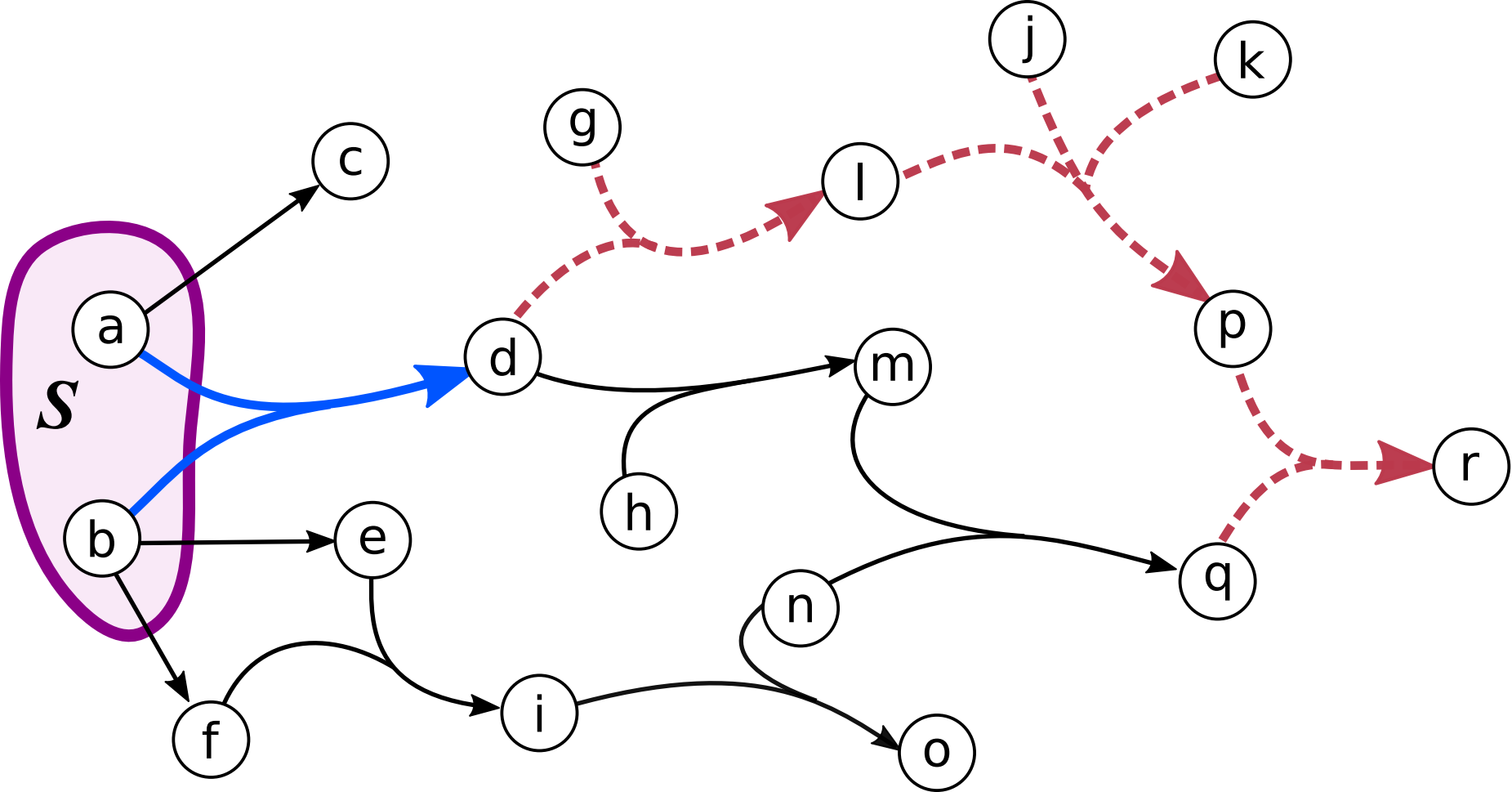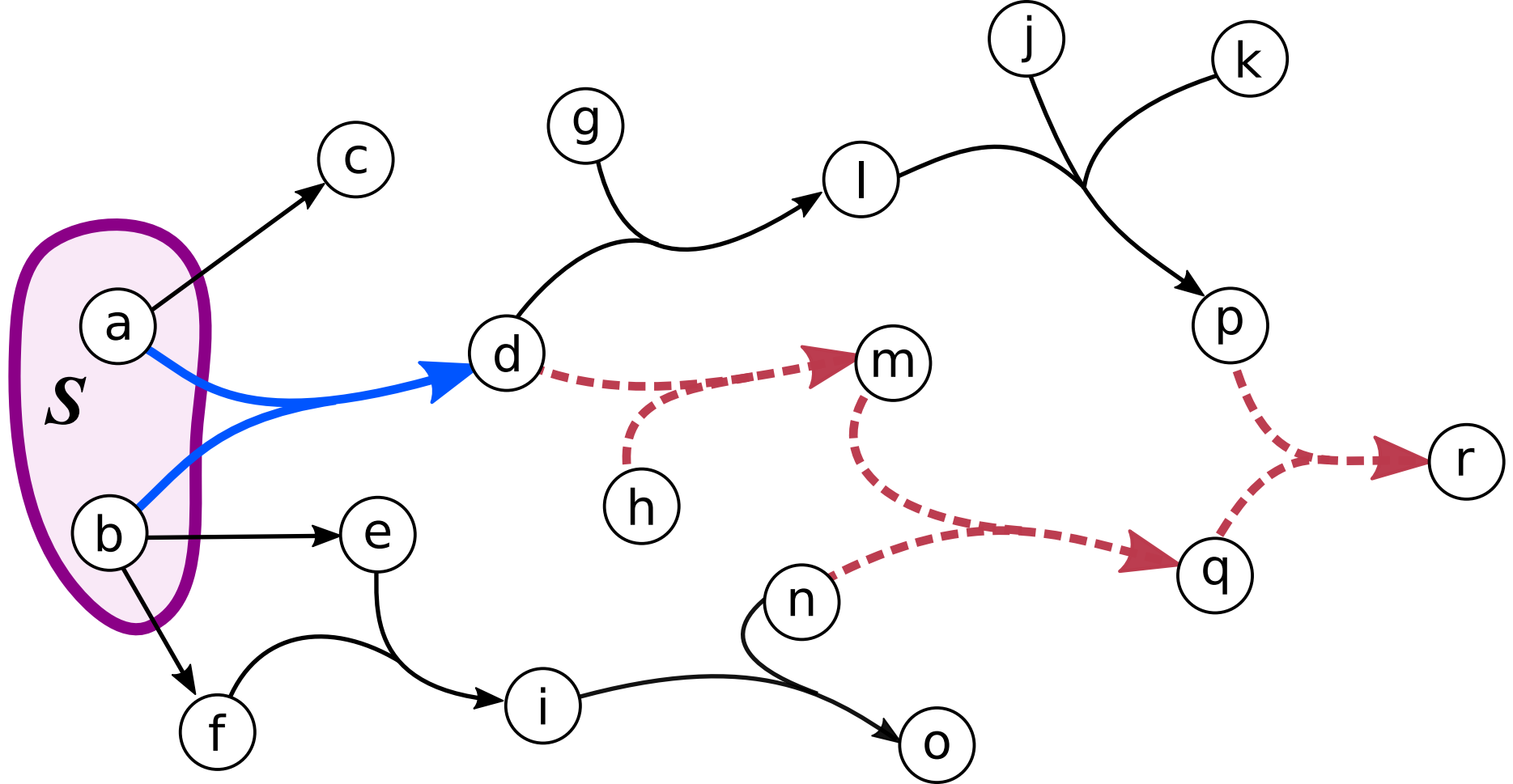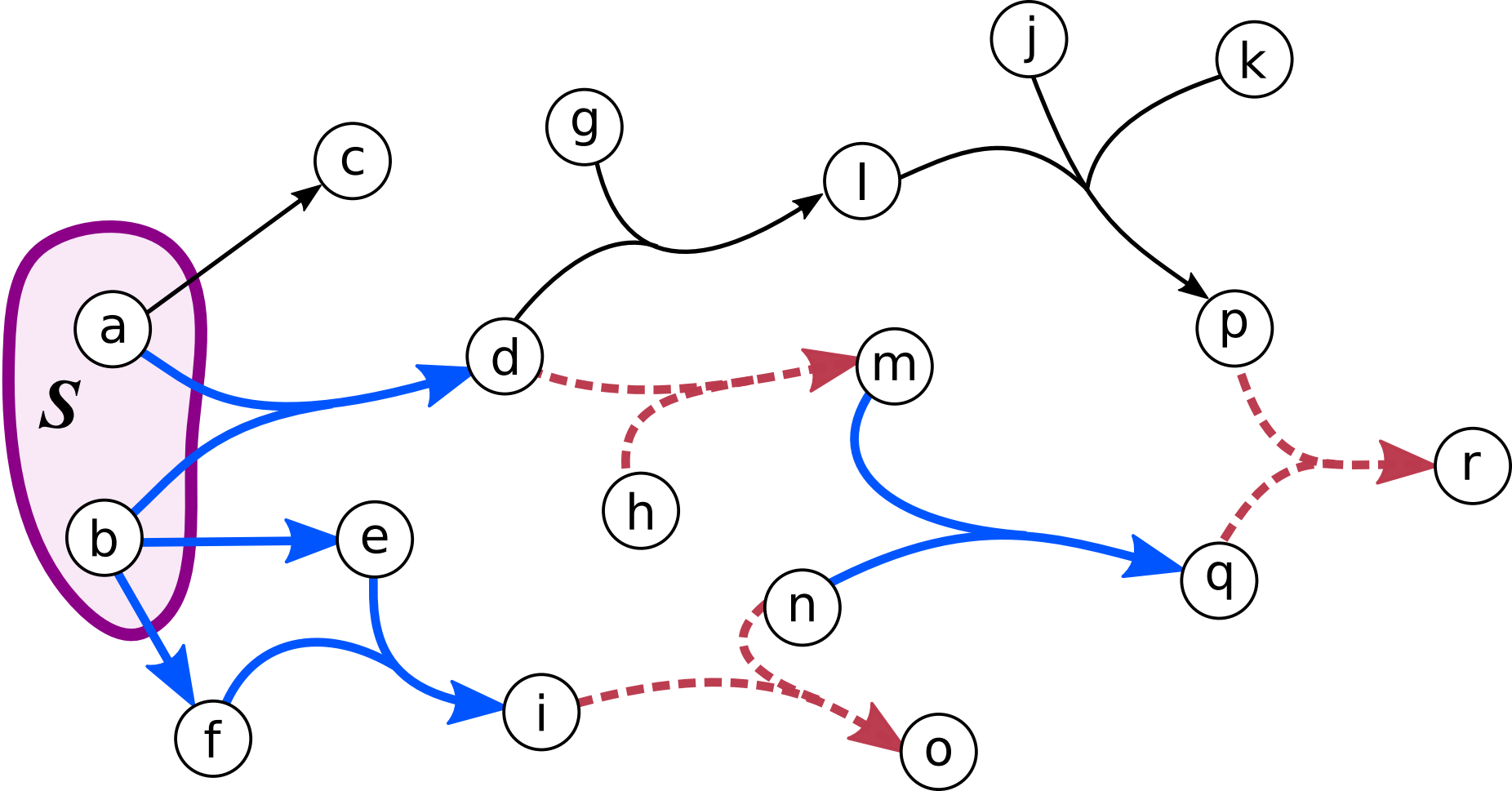

Supplement: S11 Fig — Examples of connectivity from S = {a, b} to r with a B-relaxation distance of three. Blue hyperedges denote traversals that are consistent with B-connectivity; red hyperedges denote traversals where one, but not all, nodes in the tail are connected; only hyperedges that are involved in the connectivity from S to r are highlighted for simplicity. Note that while B-relaxation distance is three, there are different sets of hyperedges that achieve this B-relaxation distance. (PDF) [file pcbi.1007384.s011.pdf]
