# Supplementary material for: Hypergraph-based connectivity measures for signaling pathway topologies
Source: PLoS Comput Biol. 2019 Oct 25;15(10):e1007384. doi: 10.1371/journal.pcbi.1007384 (PMC6834280; doi:10.1371/journal.pcbi.1007384)
Supplement: S3 Fig — Influence scores of pairs of Reactome pathways for the hypergraph at selected values of B-relaxation distance k. Rows indicate the source pathway PS and columns indicated the target pathway PT. Color indicates influence score and circle size indicates significance by permutation test (larger circles are more significant). Three selected distances are enlarged (note s3 is also in the main manuscript). (PDF) [file pcbi.1007384.s003.pdf]

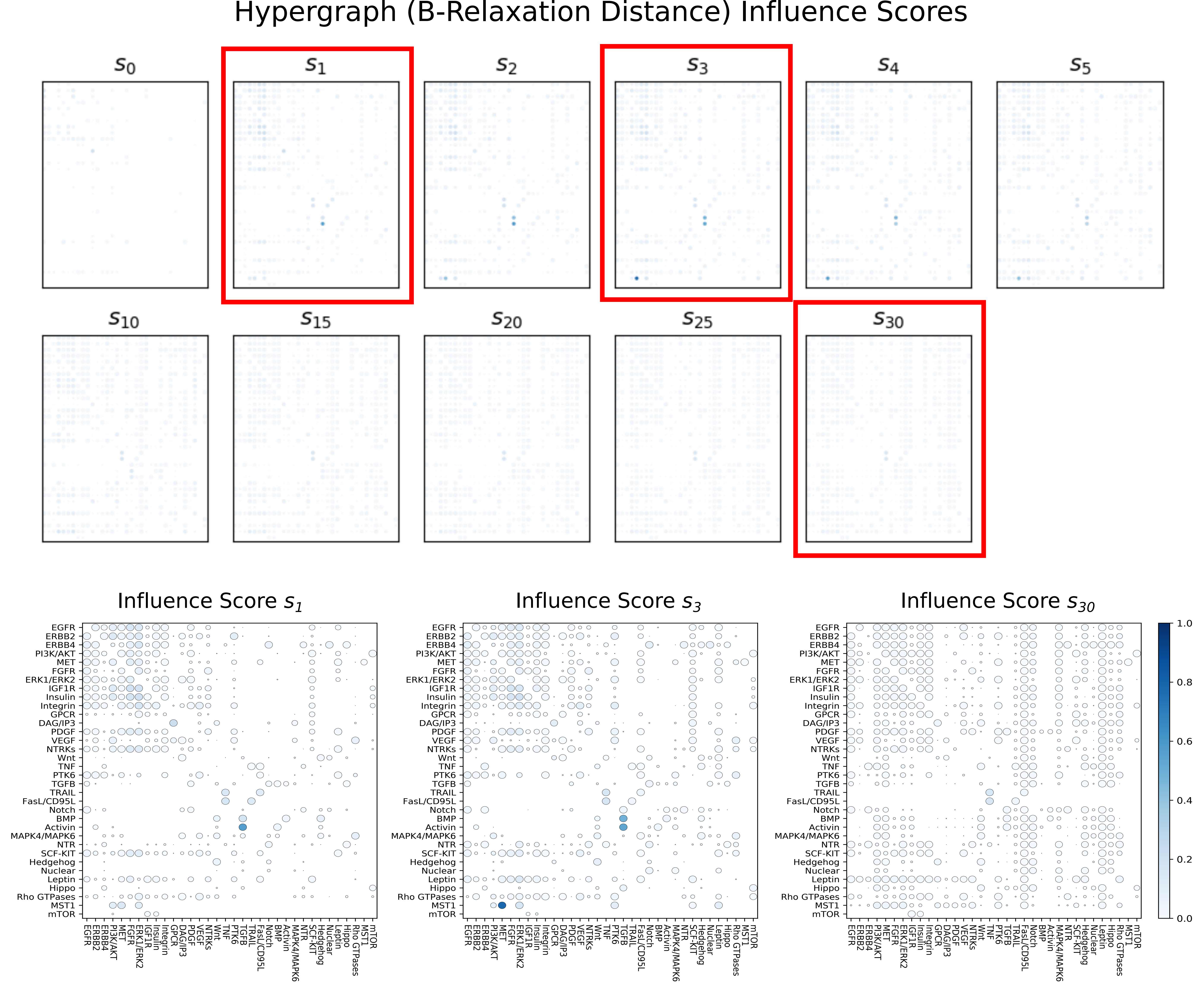

Hypergraph (B-Relaxation Distance) Influence Scores
s_0
s_1
s_2
s_3
s_4
s_5
s_{10}
s_{15}
s_{20}
s_{25}
s_{30}
Influence Score s_1
Influence Score s_3
Influence Score s_{30}
EGFR
ERBB2
ERBB4
PI3K/AKT
MET
FGFR
ERK1/ERK2
IGF1R
Insulin
Integrin
GPCR
DAG/IP3
PDGF
VEGF
NTRKs
Wnt
TNF
PTK6
TGFB
TRAIL
FasL/CD95L
Notch
BMP
Activin
MAPK4/MAPK6
NTR
SCF-KIT
Hedgehog
Nuclear
Leptin
Hippo
Rho GTPases
MST1
mTOR
1.0
0.8
0.6
0.4
0.2
0.0
