# Supplementary material for: Hypergraph-based connectivity measures for signaling pathway topologies
Source: PLoS Comput Biol. 2019 Oct 25;15(10):e1007384. doi: 10.1371/journal.pcbi.1007384 (PMC6834280; doi:10.1371/journal.pcbi.1007384)
Supplement: S1 Table — Members that are not part of any hyperedge are ignored from the hypergraph. The filtered hypergraph has removed all small molecules, two forms of Ubiquitinase, and the Nuclear Pore Complex from the hyperedges. (PDF) [file pcbi.1007384.s012.pdf]

S1 Table – Hypergraph-based Connectivity Measures for Signaling Pathway Topologies

Signaling Pathway	Reactome ID	# in Pathway	# in Hypergraph	# in Filtered Hypergraph
EGFR	R-HSA-177929	164	110	101
ERBB2	R-HSA-1227986	220	118	105
ERBB4	R-HSA-1236394	181	101	92
PI3K/AKT	R-HSA-1257604	753	357	333
MET	R-HSA-6806834	240	130	121
FGFR	R-HSA-190236	373	232	221
ERK1/ERK2	R-HSA-5684996	656	310	291
IGF1R	R-HSA-2404192	160	75	64
Insulin	R-HSA-74752	207	90	76
Integrin	R-HSA-9006921	88	66	53
GPCR	R-HSA-372790	2456	1006	796
DAG/IP3	R-HSA-1489509	107	50	32
PDGF	R-HSA-186797	244	88	80
VEGF	R-HSA-194138	351	215	183
NTRKs	R-HSA-166520	362	205	184
Wnt	R-HSA-195721	921	358	326
TNF	R-HSA-75893	127	97	93
PTK6	R-HSA-8848021	187	122	114
TGFB	R-HSA-170834	247	179	166
TRAIL	R-HSA-75158	17	13	13
FasL/CD95L	R-HSA-75157	10	10	10
Notch	R-HSA-157118	505	286	271
BMP	R-HSA-201451	74	38	36
Activin	R-HSA-1502540	44	30	28
MAPK4/MAPK6	R-HSA-5687128	200	113	105
NTR	R-HSA-193704	245	141	129
SCF-KIT	R-HSA-1433557	122	86	79
Hedgehog	R-HSA-5358351	417	190	172
Nuclear	R-HSA-9006931	431	261	208
Leptin	R-HSA-2586552	54	33	31
Hippo	R-HSA-2028269	79	47	45
Rho GTPases	R-HSA-194315	808	330	291
MST1	R-HSA-8852405	21	12	9
mTOR	R-HSA-165159	111	67	57

Reactome signaling pathways considered for pathway influence analysis. Members that are not part of any hyperedge are ignored from the hypergraph. The filtered hypergraph has removed all small molecules, two forms of Ubiquitinase, and the Nuclear Pore Complex from the hyperedges.
