# Supplementary material for: Hypergraph-based connectivity measures for signaling pathway topologies
Source: PLoS Comput Biol. 2019 Oct 25;15(10):e1007384. doi: 10.1371/journal.pcbi.1007384 (PMC6834280; doi:10.1371/journal.pcbi.1007384)
Supplement: S2 Table — We ignore the “neighbor-of” binary relation. (PDF) [file pcbi.1007384.s013.pdf]

**S2 Table** – Hypergraph-based Connectivity Measures for Signaling Pathway Topologies

| Binary Relation | # of Relations in Reactome | Conversion Rule |
|---|---|---|
| catalysis-precedes | 263,342 | Directed Edge |
| chemical-affects | 15,473 | Directed Edge |
| consumption-controlled-by | 8,079 | Directed Edge |
| controls-expression-of | 3,730 | Directed Edge |
| controls-phosphorylation-of | 3,184 | Directed Edge |
| controls-production-of | 8,052 | Directed Edge |
| controls-state-change-of | 113,558 | Directed Edge |
| controls-transport-of | 5,232 | Directed Edge |
| controls-transport-of-chemical | 5,105 | Directed Edge |
| in-complex-with | 140,302 | Undirected Edge |
| reacts-with | 1,922 | Undirected Edge |
| used-to-produce | 5,888 | Directed Edge |

**Rules for converting SIF binary relations to directed edges.** We ignore the "neighbor-of" binary relation.
