# Supplementary material for: Hypergraph-based connectivity measures for signaling pathway topologies
Source: PLoS Comput Biol. 2019 Oct 25;15(10):e1007384. doi: 10.1371/journal.pcbi.1007384 (PMC6834280; doi:10.1371/journal.pcbi.1007384)
Supplement: Attachment — Submitted filename: ResponseToReviewers.pdf [file pcbi.1007384.s015.pdf]

Response to Reviewers

Hypergraph-based connectivity measures for signaling pathway topologies
(*formerly* Connectivity measures for signaling pathway topologies)
Nicholas Franzese, Adam Groce, T. M. Murali, and Anna Ritz[1]

We thank the reviewers for their careful reading of our manuscript. We address Reviewer #1 & #2's minor edits together and then address each of Reviewer #3's comments in a point-by-point response. Our responses below are in italics, and we highlight changes to the manuscript in blue (changes to the supplement are not highlighted).

Note that we have changed the title from "Connectivity measures for signaling pathway topologies" to "Hypergraph-based connectivity measures for signaling pathway topologies." This is due to an earlier comment from a reviewer after the Great Lakes Bioinformatics (GLBio) submission, who noted that "the title is very broad and suggests that this paper is a review." While we comprehensively assess connectivity measures for four representations, we decided to change the title since our main contribution is a relaxation of hypergraph-based connectivity. We hope that the title change is acceptable during this revision.

1 Reviewers #1 and #2

Reviewer 1: The authors have addressed my comments fully in the revised manuscript and response to reviewers. A very nice paper!

Reviewer 2: I was a reviewer for the conference version of the paper to GLBIO. The authors have done a great job in their response to reviewers. The manuscript is in great shape, and I don't have any major comments.

Thanks for your initial comments in the GLBio submission, they really improved the manuscript.

1.1 Minor Comments

Both Reviewers #1 and #2 had minor typographical and cosmetic comments. We have combined them in this section.

1. The type size in the compiled manuscript in all figures except for 4 is too small. If possible, it would be nice to standardize the font size across the figures.

   We have made the following adjustments to ease readability of the figures, and we will work with the editors to resize the figures if necessary.

   Fig1*: increased the font of the smallest words.*

   Fig2*: Reduced whitespace around figures; narrowed the width of the figure so text will appear larger.*

   Fig3*: Increased the font side of the labels; reduced whitespace around figures.*

   Fig5*: Moved the labels inside the subfigure boundaries; reduced whitespace around figures.*

   Fig7*: Enlarged numbers in Venn diagrams; widened bottom plot in Panel A.*

   Fig10*: rearranged layout so images appear larger; removed third-party logos.*

   Unfortunately, Fig6 cannot be enlarged – however we provide links to the GraphSpace graph for further exploration. Figure 4 is modified in the next comment, and Figures 8 & 9 are addressed in a comment by Reviewer #3.

2. Figure 4A: Is it possible to stretch A so that one can look at the same pathway across both A and B?

   This is fixed.

[1]Corresponding author: aritz@reed.edu

3. Figure 4B: What is the threshold for significance? Are the p-values corrected for multiple testing?

   Our threshold for significance was the case where all 1,000 permutation tests had influence scores strictly less than the observed influence score (corresponding to 0 permutations with a score that was equal to or greater than the observed score). Eighty-four tests (about 7%) passed this threshold, and we discuss the three pathway pairs that have large influence scores for $k = 3$ (the dark, large circles in Fig. 4). The p-values were not corrected for multiple testing. We have clarified our definition of significance with the following sentences:

   "We computed s_k for every pair of Reactome signaling pathways for every value of k (Supplementary Fig. S3 and the Supplementary Dataset). We say a pathway pair's influence score is significant if there are no permutation tests (out of 1,000) with a score greater than or equal to the observed score. There are three significant pairs that exhibit a large influence score for $k = 3$ (large, dark circles in Fig. 4B)..."

   We have also added a legend to Fig. 4 that describes the circle size, and we also provide an Excel file with this data (see the last section, under "Additional Questions").

4. In the Abstract: "pairs of pathways statistically significant influence" → "pairs of pathways with statistically significant influence"

   This is fixed.

5. Page 3, line 43: k hasn't actually been defined before this point

   We have modified the sentence to be: "...while these relationships appear in the bipartite graph representation, they only emerge further away from the upstream 'source' pathway."

6. Page 4, line 68: "built upon" → "encoded within"?

   Thanks for the suggestion, we changed it.

7. Page 6, lines 145-162: I got a bit confused re-reading this section. The text describes three sets of nodes to remove: 2778 small molecules defined by Reactome, 3 highly connected gene products/complexed, and another 155 small molecules from PathwayCommons. Which of these are "blacklisted nodes" – the ubiquitins and NPC? Were both sets of small molecules combined into one? Do the stats on the numbers of affected nodes/hyperedges reflect the removal of all three sets of nodes?
   (related comment): I think that the cross-refs for Fig 3B and 3C might be flipped in the text.

   The cross-references for Fig 3B and 3C are correct; they were presented out of order in the text (Fig 3C before 3B) because of the visual of deceasing connectivity of Fig 3 A-B-C. We have rearranged this paragraph to be more clear and to describe Figure 3B before figure 3C. It now reads as follows:

   PathwayCommons reports ubiquitous molecules in their database ("blacklisted nodes" [19]), and 155 of these appear in the hypergraph. Removing these molecules from the hypergraph reduces the number nodes that are connected to many others (Fig. 3B). However, we found that small molecules remained even after removing these "blacklisted" nodes. Instead of using the PathwayCommons list, we pruned the hypergraph by removing the 2,778 nodes labeled as small molecules by Reactome, as well as three other highly-connected entities (cytosolic Ubiquitin, nuclear Ubiquitin, and the Nuclear Pore Complex). In total, we altered 5,180 hyperedges by removing these 2,781 entities, resulting in a filtered hypergraph with 15,440 nodes and 8,773 hyperedges. In this hypergraph, even fewer nodes are connected to many others, and the transition from low-to-high connectivity is more gradual across different source nodes (Fig. 3C).

8. Page 10, line 242: "under any definition" – definition of what?

   We have modified the sentence to be: "Under any definition of connectivity, two connected proteins may have a functional relationship rather than a physical one."

9. Page 11, line 23: "pathwa" → "pathway"

   This is fixed.

10. Page 11, line 307: "Reactome pathwa" → "Reactome pathway"

    We could not find another instance of "pathwa" without the "y" besides the previous typo.

2 Reviewer #3

As a new reviewer for this revised submission, these comments are intended to assess only the responsiveness of the revisions to the major concerns from the original reviewers. The major changes in the revision include several new analyses that better demonstrate how B-relaxation distance can be used in practice and how this distance measure relates to existing connectivity measures. The new figures showing reachable source-target paths as a function of distance satisfy prior concerns about how the parameterized B-relaxation measure relates to parameterized versions of other connectivity measures.

Several reviewers questioned how B-relaxation can be evaluated empirically with more comprehensive applications. The new evaluation of STRING edges by score and edge type is a strong response to these comments. The results depict how pairs of proteins that are B-connected or have a particular B-relaxation distance tend to have higher STRING edge scores, especially for the STRING combined scores. This emphasizes how B-relaxation provides a different way to evaluate node connectivity that is not trivially recapitulated by the existing connectivity measures or Reactome pathway membership. The reviewers noted several additional potential applications that the authors defer for future work. This is reasonable to keep outside the scope of this manuscript and shows the opportunities for future studies based on B-relaxation connectivity. In addition, the authors added new permutation-based statistical testing for their Reactome pathway influence analysis. This is an appropriate control because the authors are correct that simulating realistic pathway hypergraphs, a previously-suggested alternative way to test the influence measures, is non-trivial.

The minor comments regarding the precision and recall analysis for the pathway influence study would have been a helpful addition to the manuscript. However, this is a fairly minor request and would not substantially affect the conclusions about the merits of B-relaxation.

Overall, all major concerns have been directly or indirectly addressed. The GraphSpace network visualizations and software to reproduce the major analyses in the manuscript are added benefits that complement the strong manuscript. The specific comments below are all minor comments that could help improve the presentation.

We thank the new Reviewer for their positive comments as well as their suggestions to improve the manuscript.

2.1 Minor Comments

1. Reactome has a large number of pathways, but only 34 are used for the pathway influence analysis and 140 are used for the STRING edge score analysis. It is unclear why these subsets where selected and why different pathways were used for the influence and STRING analyses.

   We did not clearly describe or motivate the selection of Reactome pathways for our experiments. Pathways are organized hierarchically within Reactome (see, for example, details about the Pathway Browser in the user guide (`https://reactome.org/userguide/pathway-browser`). We have added a subsection in the Methods that describes how we use the Reactome pathway hierarchy to select pathways (see below). We have also added references to this subsection when we describe the Reactome pathways in the Results.

   Reactome pathways used for influence analysis and STRING benchmarking

   We consider 34 Reactome pathways for assessing pathway influence across reactome. Pathways are organized hierarchically within Reactome, and the 34 pathways were labeled as direct children of "Signal Transduction" and "Signaling by Receptor Tyrosine Kinases" in the Reactome file provided by PathwayCommons v10 (Supplementary Table S1).

   When we benchmark functional relationships using STRING evidence channels, we expand the set of Reactome pathways to include others not annotated to "Signal Transduction." We identified 140 non-redundant pathways for this analysis as follows. We first collected entities annotated to each Reactome pathway and sub-pathway, and then removed the 26 "top-level" pathways in the Reactome hierarchy from consideration (Cell Cycle, DNA Repair, Immune System, etc.). These "top-level" pathways are general and are represented by more specific sub-pathways in the list. We finally removed all sub-pathways that are completely contained in another pathway. These redundant pathways do not add any additional pairwise relationships to the benchmarking analysis.

2. The STRING analysis excluded the "database" channel, which used Reactome as a source of evidence. However, if the "combined score" channel includes the "database" channel as an information source, there is still some degree of circularity in this analysis.

   We agree that this was not noted; however, we believe that the "combined score" channel is still useful for benchmarking. We have rearranged the paragraph that describes the STRING channels we use, and we have added the following sentence: While the "combined score" channel includes information from Reactome, only 6% of the edges from this set have evidence from the "database" channel.

3. The figures for the STRING analysis show scores in the 0-1000 range. However, some of the text describes scores in the 0-1 range.

   Thanks for the catch regarding the inconsistent scoring. STRING reports channel scores in the 0-1000 range, but when they combine scores they use a 0-1 scale. We have adjusted all plots in Figure 7 and the supplement to be on a scale between 0-1 to avoid confusion.

4. Using 2.2e-308, the smallest float in Python, in the STRING significance analysis may not be the best way to present these results. Wolfgang Huber discussed this issue recently in "Reporting p Values".

   Thank you for point us to this paper - it does summarize your comment nicely. We have added this paper to the references and adjusted the text in the citation of Figure 7 to be: the violin plot shows the distributions of interaction scores (which range from 1 to 1000) for different sets of interactions (median, percentiles, and Kruskal-Wallis p-values shown, where $p < 2.2\mathrm{e}{-16}$ is below the detection limit [43].

5. In Figure 8 and other figures that visualize hypergraph properties, it is not immediately obvious that the blue regions overlap the red regions. Even if the figures are not changed, it would be helpful to explicitly note in the caption that node d is in both the blue and the red sets.

   We thank the reviewer for pointing out this potential confusion. The regions in Figure 8 denote sets of hyperedges (rather than nodes). Further, it is even less clear in Figure 9 that the blue regions blue regions denote sets of nodes while the red regions denote sets of hyperedges. We have modified these figures (as well as supplemental figures) to clearly denote whether the sets denote nodes or hyperedges (an example of Figure 8 is shown below).

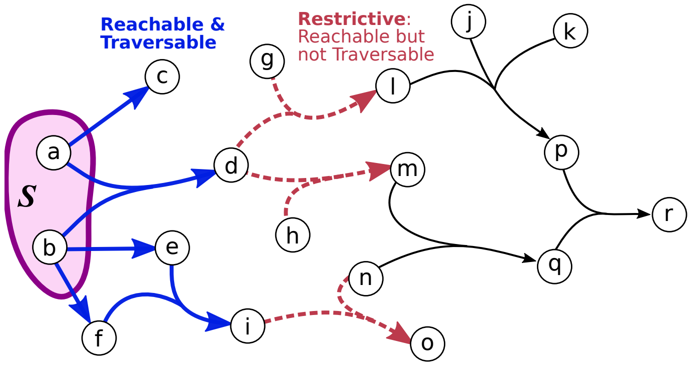

6. The Discussion notes that connected proteins that are not in the same pathway may be missed from gene-set-like enrichment approaches. For most applications of gene set enrichment, this seems like a desirable property.

   We have modified the sentence to read: "We found that many interactions are connected in Reactome but are not annotated to the same Reactome pathway, thus these potential downstream effects may be missed from gene-set-like enrichment approaches."

7. The Python software is provided in `https://github.com/Murali-group/halp` and `https://github.com/annaritz/pathway-connectivity` and is well-organized in general. However:

   - The GPL3 licenses are not stated directly in the manuscript.
     We have added the GPL-3.0 licenses for HALP and the `pathway-connectivity` *GitHub repo to the main manuscript and the supplementary information.*

- It is not clear which developmental branch of the halp repository was used for this study, which impedes reproducibility. Ideally this version would be merged to master and released.

  The developmental branch is called `annabranch` *– we agree that it should be merged with the master and released. We are working with the team do merge this branch and release a new version of halp. We plan to release a new version of* `halp` *with an implementation of B-relaxation distance upon publication.*

- There are test cases, but the Travis CI testing fails.

  We are having some package install issues with Travis CI testing, but all unit tests pass on the `annabranch` *branch (which include tests for B-relaxation distance). We will continue to work on this and update the GitHub repo.*

```
/path/to/halp/$ pytest -x
===== test session starts =====
platform darwin -- Python 3.5.1, pytest-3.7.1, py-1.5.4, pluggy-0.7.1
rootdir: /path/to/halp, inifile:
collected 120 items

tests/test_directed_hypergraph.py ................................   [ 26%]
tests/test_undirected_hypergraph.py ..................               [ 41%]
tests/algorithms/test_directed_paths.py .......................      [ 60%]
tests/algorithms/test_directed_random_walk.py .                      [ 61%]
tests/algorithms/test_k_shortest_hyperpaths.py .............         [ 72%]
tests/algorithms/test_undirected_partitioning.py ...                 [ 75%]
tests/utilities/test_directed_graph_transformations.py ...           [ 77%]
tests/utilities/test_directed_statistics.py .......................  [ 96%]
tests/utilities/test_priority_queue.py .                             [ 97%]
tests/utilities/test_undirected_graph_transformations.py ...         [100%]
===== 120 passed in 1.17 seconds =====
```

- The pathway-connectivity repository contains data from third-party sources (e.g. STRING) that may require more direct attribution in the readme.

  We now fully attribute STRING, PathwayCommons, and the BioPAXSTREAM code. We did a major overhaul of the scripts contained in the repo – there is now a single-point `run.py` *script along with all required datasets. In addition, we are adding the in-house parser scripts to generate the representations (graphs, compound graphs, hypergraphs, and bipartite graphs) – graphs and compound graphs are already added, and the remaining representations will be complete by publication. We will continue to clean up this repo to make it more usable.*

- An added bonus for both repositories would be to archive the software on third-party services like Zenodo, Figshare, or Software Heritage to ensure they are more permanently available to readers.

  Thanks for this idea – we will consider archiving the software upon publication, once all code to generate the data is up.

8. Typos and phrasing:

- Abstract: "pathways statistically significant" is missing a word

  This now reads "pathways with statistically significant..."

- P3: "molecules that that participate"

  This is fixed.

- P3: The end of the introduction notes relationships that "emerge for larger values of k" but the parameter k has not yet been introduced and is not intuitive before that introduction

  We thank the reviewer for this catch. We have modified the sentence to be: "...while these relationships appear in the bipartite graph representation, they only emerge further away from the upstream 'source' pathway."

- P9: "nodes that in"

  This now reads "nodes that are in"

- P11: "within the same Reactome pathwa"

  This is fixed.

- P16: “PaxTools java” could capitalize Java
  This is fixed.

Additional Questions

Have all data underlying the figures and results presented in the manuscript been provided?

Reviewer #1: No: The statistics underlying the figures are not provided as supplementary tables, but the authors have provided their input data and code to generate the results on GitHub.

Reviewers #2 and #3: Yes

Yes, all data and statistics can be reproduced using the code and inputs described on the GitHub site. In addition, we now include an Excel table of the pathway influence scores for hypergraph B-relaxation distance that reflects the values in Fig. 4 and Supplementary Fig. S3. The sheets include the Jaccard pathway overlap and, for multiple values of k, the influence scores s_k *and the proportion of permutations that achieved a score greater than or equal to the observed score.*
